# Single Cell Analysis Reveals Dynamic Changes of Distinct Cell Populations in Human Nickel Allergy

**DOI:** 10.1111/all.70108

**Published:** 2025-10-15

**Authors:** Marc Schmidt, Andrea Knorz, Katharina Meder, Simon Goller, Fabian Imdahl, Yamila Rocca, Matthias Goebeler, Pierre Khoueiry

**Affiliations:** ^1^ Department of Dermatology, Venereology and Allergology University Hospital Würzburg Würzburg Germany; ^2^ Single Cell Center Würzburg Helmholtz Institute for RNA‐Based Infection Research (HIRI) Würzburg Germany

**Keywords:** allergic contact dermatitis, innate immune activation, nickel allergy, single cell analysis, T cell response

## Abstract

**Background:**

Metal allergies are prime examples of delayed‐type hypersensitivity divided into two phases: in the sensitization phase, initial contact with an allergen leads to activation of skin‐resident cells and formation of metal‐reactive T cells. during elicitation, these T cells mount an immune response resulting in clinically apparent eczema within 72 h after exposure. Two main mechanisms have been implicated in the initiation of metal hypersensitivity: direct or indirect activation of innate immune receptors such as Toll‐like receptor 4, and conditional innate immune activation via the NLRP3 inflammasome. Yet, the responsible cell type(s) mediating these responses are unknown. Moreover, it is unclear whether the elicitation phase is mainly dominated by infiltration of circulating metal‐responsive T cells or if tissue‐resident T cells contribute.

**Methods:**

Here, we analyzed the relevance of different cell types in human nickel hypersensitivity by single‐cell RNA sequencing and immunofluorescence analysis of skin samples of nickel‐sensitized donors epicutaneously exposed to diluent and nickel for 8 or 72 h.

**Results and Conclusion:**

Nickel specifically activated distinct populations of endothelial cells, suprabasal keratinocytes, fibroblasts, and *CCR7* ^*+*^ dendritic cells, co‐expressing the TLR4‐interacting proteogylcan *DCN* and CCR7 ligand *CCL21* within 8 h. Skin‐resident T cells were not involved in the early hypersensitivity response, as their gene expression remained unaltered 8 h after nickel exposure. However, substantial changes in the cutaneous T cell compartments occurred after 72 h, with massive infiltration of KLF2 ^+^ central memory T cells being a recurrent feature of both nickel‐sensitized patients and individuals allergic to the glucocorticoid contact allergen budesonide.

## Graphical abstract

We investigated cell types mediating the early and late inflammatory responses in human Ni^2+^‐allergy by scRNA‐Seq. Early responsiveness was mainly mediated by TLR4^+^TAGLN^−^ endothelial cells indirectly activating TLR4^−^KRT16^+^ keratinocytes, and CCR7^+^DCN^+^ DCs. Late responsiveness was crucially driven by infiltration of KLF2^+^ central memory T cells and TLR4^+^CD163^+^CCR7^−^ DC3 cells. CCR7, C‐C chemokine receptor type 7; DC, dendritic cells; EC, endothelial cells; IF, immunofluorescence; KC, keratinocytes; KLF2, Krüppel‐like factor 2; Ni^2+^, nickel; scRNA‐Seq, single cell RNA sequencing; SELL, L‐selectin; TAGLN, transgelin; T_CM_, central memory T cell; Th, T helper cell; TLR4, Toll‐like receptor 4.

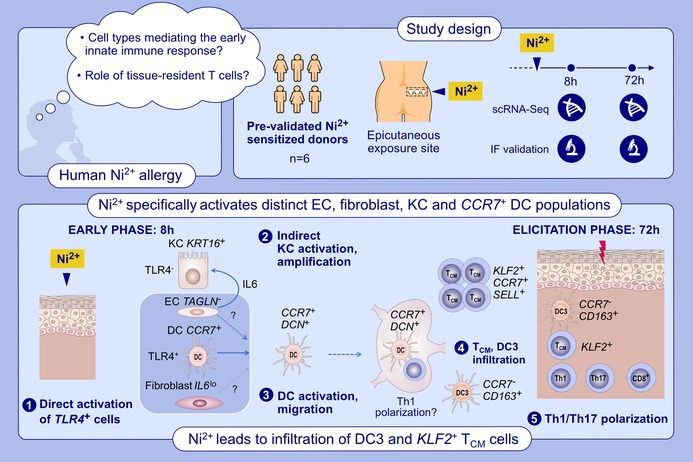

**Abbreviations**
ACD: allergic contact dermatitis
CHS: contact hypersensitivity
Cr_2_O_7_^2−^
: dichromate
DC: dendritic cell
DEG: differentially expressed gene
EC: endothelial cell
EndMT: endothelial‐to‐mesenchymal transition
HTO: hashtag oligonucleotide
KC: keratinocyte
Ni^2+^
: nickel
NK: natural killer cell
mregDC: mature DC enriched in immunoregulatory molecules
PCA: principal components analysis
scRNA‐Seq: single cell RNA sequencing
SMC: smooth muscle cell
T_CM_
: central memory T cell
T_RM_
: Skin‐resident memory T cells
UMAP: Uniform Manifold Approximation and Projection
UMI: unique molecular identifier

## 1 Introduction

Contact hypersensitivity (CHS) is a classical model for T cell‐mediated delayed‐type hypersensitivity [1, 2]. In humans, the most relevant CHS is allergic contact dermatitis (ACD) to the metal ion nickel (Ni^2+^), released from everyday products such as jewelry, coins, implants, or mobile phones [2, 3]. Moreover, allergy to dichromate (Cr_2_O_7_^2−^) used in the leather industry or the construction sector accounts for a significant portion of occupational ACD [2].

Like all CHS responses, metal‐induced ACD occurs in two phases [2, 3]: In a clinically inapparent sensitization phase, metal‐reactive T cells develop. Upon elicitation, these T cells mount an adaptive immune response at the exposure site manifesting as pruritic eczema 48–72 h after challenge.

A key requirement for metal‐induced ACD is innate immune activation [4, 5] occurring by two major mechanisms [6]:

1. Direct activation of pattern recognition receptors (e.g., TLR4 by Ni^2+^ and cobalt [7, 8]), or their indirect activation by damage‐associated molecular patterns [4, 5].
2. Conditional innate immune activation via the “inflammasome” protease complex as reported for Cr_2_O_7_^2−^ that triggers IL‐1β release via the NLRP3 inflammasome upon appropriate TLR priming [9].

For both classes of metal allergens, the key cell type(s) mediating the required innate immune response are unknown. In human Ni^2+^ allergy, keratinocytes (KCs) rapidly produce the cytokine CCL2 [10]. However, KCs lack significant *TLR4* or *NLRP3* mRNA expression, rendering cultured KCs insensitive to Ni^2+^‐ or Cr_2_O_7_^2^ ‐induced innate immune activation [11]. Moreover, a recent study suggests a suppressive role of KCs in effector T cell function in human Ni^2+^ allergy [12], raising questions regarding their exact role in the early phase of metal allergy. It further remains unknown whether innate immune activation may differ in sensitized versus non‐sensitized individuals due to potential presence of metal‐responsive tissue‐resident memory T cells (T_RM_). The latter have been implicated in mediating CHS by model allergens in mice with some evidence indicating a similar role of T_RM_ in humans [13, 14, 15].

To address these questions, we performed single‐cell RNA sequencing (scRNA‐Seq) of skin cells from donors sensitized to Ni^2+^ but not the unrelated metal allergen Cr_2_O_7_^2−^.

## 2 Methods

### 2.1 Patient Selection and Experimental Design

Three Ni^2+^‐sensitized, Cr_2_O_7_^2−^‐negative (Ni^2+ pos^, Cr_2_O_7_^2‐ neg^) human donors were epicutaneously exposed on the hip to a diluent (vaseline control) for 8 h or to 5% NiSO_4_ for 8 h and 72 h using occlusive Finn chambers. Additionally, one donor received 0.5% K_2_Cr_2_O_7_ for 8 h and 72 h as further negative control. Punch biopsies (6 mm for 8 h, 8 mm for 72 h) were collected and processed for multiplexed scRNA‐Seq, as detailed in the Supporting Information. To control batch effects and confirm reactivity, 72 h samples were stimulated 64 h before the 8 h samples (Figure S1).

For immunofluorescence analysis, cryomaterial from three independent Ni^2+ pos^, Cr_2_O_7_^2‐ neg^ donors or a patient sensitized for the glucocorticoid allergen budesonide was obtained, stored in liquid nitrogen, and stained as outlined in the Supporting Information. Biopsies were collected with informed consent, with approval of the study protocol by the University of Würzburg ethics committee (No. 19/22‐sc).

### 2.2 ScRNA‐Seq Dataset Pre‐Processing and Analysis

FASTQ files from Illumina BCL were processed using 10× Genomics Cell Ranger 7.0.1 or 8.0.1 for quality control, mapping to GRCh38, barcode processing, and unique molecular identifier (UMI) counting. Background noise was removed with CellBender [16] for non‐Hashtag Oligonucleotide (HTO)‐generated samples and single‐cell analysis conducted using Seurat (v5) [17]. Cells with <200 genes or > 10% mitochondrial UMIs were filtered out and doublets excluded using scDblFinder v1.14 [18] for non–HTO‐generated samples. For samples generated using HTO libraries, expression matrices were normalized using the log normalization method (scale factor = 10,000) and “centered log ratio” method. Data were scaled and principal component analysis (PCA) was performed. Integration of all samples was done using Harmony v1.2.3 [19], followed by RunUMAP for dimensionality reduction. Clustering was performed with FindNeighbors and FindClusters using Harmony embeddings. Quality was assessed by density plotting of genes/UMI, with values > 0.8 log10 considered acceptable. Differentially expressed genes (DEGs) were identified with FindAllMarkers (Wilcox test, min.pct = 0.2, logfc.threshold = 0.25). Cell type annotations were manually determined through iterative inspection of differentially expressed canonical markers and publicly available scRNA‐Seq data from the Protein Atlas database (https://www.proteinatlas.org/). The final resolution chosen was 1.2. Frequencies of cells/condition were plotted with dittoSeq [20] and statistical significance assessed using chi‐squared tests with Bonferroni adjustment. Interaction between different cell subsets was analyzed using CellChat [21] and trajectory analysis performed using pseudotime [22]. Further details are provided in the Supporting Information.

## 3 Results

### 3.1 Composition of Transcriptionally Distinct Skin Cell Clusters in Human Ni^2+^ Allergy

To identify the relevant cell types and signals involved in early and late events of human Ni^2+^ allergy, we performed scRNA‐Seq on skin cells from three independent donors sensitized to Ni^2+^ but not Cr_2_O_7_^2−^. All donors were epicutaneously exposed to Ni^2+^ for 8 and 72 h, or to diluent for 8 h as reference (Figure S1). Additionally, one donor received the unrelated metal allergen Cr_2_O_7_^2−^ for 8 and 72 h as a control for an unrelated metal allergen.

**FIGURE 1:**
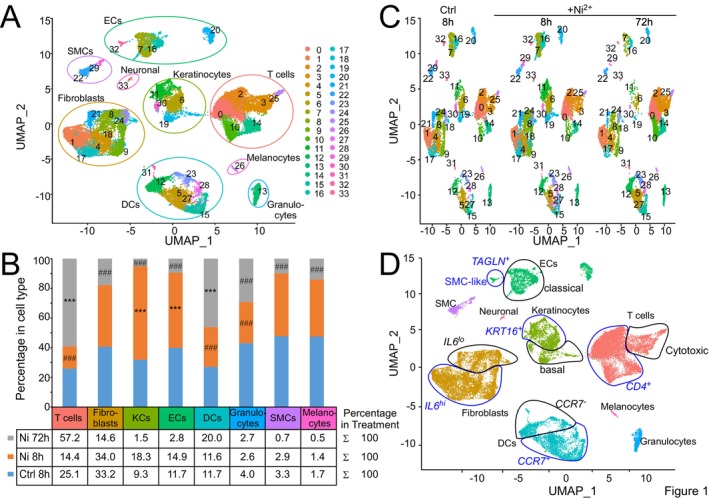
Composition and dynamic changes of transcriptionally distinct cell subsets in the skin of Ni^2+^‐sensitized donors upon epicutaneous stimulation with the contact allergen Ni^2+^. (A) Overview of the skin cell subsets present in human ACD to Ni^2+^ identified by UMAP clustering of scRNA‐Seq data. Shown is an UMAP plot of the integrated scRNA‐Seq data set of a total of 29,021 cells isolated from three Ni^2+^‐sensitized donors exposed epicutaneously to diluent for 8 h or to Ni^2+^ for 8 or 72 h. One donor additionally received exposures with the unrelated metal allergen Cr_2_O_7_^2−^ for 8 or 72 h. Data from all donors were integrated and normalized across samples. (B) Bar chart, showing the percentage distribution for each cell type identified in (A) separately, and broken down by the indicated treatment. Cell counts obtained for the Cr_2_O_7_^2−^ treatments were not taken into account. The cell type‐specific cell counts of the respective treatments were each normalized to the total cell count of the specified treatment and obtained totals for each cell type set to 100%. An additional table shows the total percentage distribution of cell types identified in the specified treatment sample. Asterisks and hash tags indicate statistically significant upregulation or downregulation of cell counts relative to control and the respective other treatment condition (***/###, *p*‐val_adj < 0.001, chi‐squared test with Bonferroni correction). (C) UMAP representation of the scRNA‐Seq data from (A) split by treatment, illustrating Ni^2+^‐dependent changes in cell subcluster composition over time. (D) UMAP of the combined sequencing dataset as in (A) but with the identified main cell types displayed in similar color and relevant subclusters highlighted with blue and black marks and labeling. Clusters were annotated by manual cell typing based on specific enrichment of selected marker genes (threshold: avg_log2 FC > 1.5, *p*‐val_adj < 0.05) expressed in the included individual cell clusters.

Computational processing of the integrated samples identified 29,021 cells in 34 clusters (Table S1) that were classified into five large groups of ≥ 4 clusters and three small groups comprising ≤ 2 clusters (Figure 1A). Cell annotation identified the five major groups as T cells, fibroblasts, keratinocytes (KC), endothelial cells (EC), and dendritic cells (DCs) (Figure 1A). The three smaller groups corresponded to smooth muscle cells (SMC), melanocytes/neuronal cells (sharing expression of the neuronal lineage marker SOX10 but separated by expression of pigmentation genes) and granulocytes. Expression analysis validated the specificity of our employed cell type markers (Figure S2).

Statistical evaluation of main cell type counts between the Ni^2+^‐exposed samples and the diluent control revealed increases of KCs and ECs after 8 h Ni^2+^ exposure, and of T cells and DCs after 72 h (Figure 1B, Table S2). Moreover, T cell and granulocyte counts were statistically reduced at 8 h Ni^2+^ exposure, albeit the former appeared unspecific and was similarly seen with Cr_2_O_7_^2−^ (Figure S4B). Direct comparison of the UMAPs identified DC cluster 27 as a Ni^2+^‐specific population almost exclusively present at the early (8 h) time point (Figure 1C). KC cluster 11, which separated into distinct subclusters at higher resolution (Figure S3), EC cluster 16, and fibroblast cluster 18, showed slightly altered shapes due to significantly increased cell counts in the 8 h Ni^2+^‐exposed sample compared to control and the 72 h sample (Figure 1C). Other obvious changes included decreased KC numbers after 72 h Ni^2+^ exposure, consistent with increased T cell infiltration and KC cytotoxicity observed during Ni^2+^‐induced ACD [23] but statistical reductions were also observed with other cell types including ECs (Figure 1B,C Table S2). None of these changes occurred in the Cr_2_O_7_^2−^‐exposed samples as also confirmed by cell type distribution analysis of the respective samples (Figure S4A,B).

### 3.2 ECs, KCs, DCs, and Fibroblasts Are the Main Early Ni^2+^‐Responsive Cell Types

To investigate changes not directly obvious by mere comparison of cell frequencies and cluster composition, we compared gene expression patterns of corresponding cell types in control and the Ni^2+^‐exposed samples. After 8 h of Ni^2+^ stimulation, only ECs, KCs, DCs, and fibroblasts showed an appreciable number of DEGs (Figure S5A). Next, we compared subsets within the five main clusters. For DCs, we discriminated *CCR7* ^*−*^ from *CCR7* ^*+*^ DCs, which marks migratory, lymph node‐homing DCs [24, 25, 26] negative for the myeloid lineage marker *ITGAM* (Figure S6A and Figure 1D). EC clusters were subdivided by *TAGLN* positivity as we noticed high expression of *TAGLN* and other SMC‐like mesenchymal genes (e.g., *CALD1*) along with a reduction of endothelial markers (e.g., *PECAM1*) in cluster 32, discriminating them from classical vascular ECs in clusters 7 and 16 and the *LYVE‐1* ^+^ *PROX‐1* ^*+*^ *CCL21* ^*+*^ lymphatic ECs in cluster 20 (Figure S6B, Figure 1D). For KCs, we chose *KRT16* for discrimination that was exclusively expressed in the suprabasal KC clusters 6 and 11 characterized by the expression of the differentiation markers *KRT1*, *IVL* and *TGM1* and lack of the basal cell marker *KRT5* (Figure S6C, Figure 1D). T cells were classified by *CD4* ^*+*^, distinguishing TH cells (clusters 0, 2, 10, 14) from cytotoxic T cells (clusters 3, 25) (Figure S6D; Figure 1D). Last, for fibroblasts, we differentiated between clusters with low (clusters 8, 18, 21, and 24) and high *IL6* expression (clusters 1, 4, 9, 17, Figure 1D, and Figure S6E). These comparisons revealed *CCR7* ^*+*^ DCs, *TAGLN* ^*−*^ ECs, suprabasal *KRT16* ^*+*^ KCs and *IL6* ^*lo*^ fibroblasts as major subsets mediating the early response to Ni^2+^ (Figure S5B).

### 3.3 Ni^2+^ Triggers Early and Late Changes in Distinct DC Subsets

**FIGURE 2:**
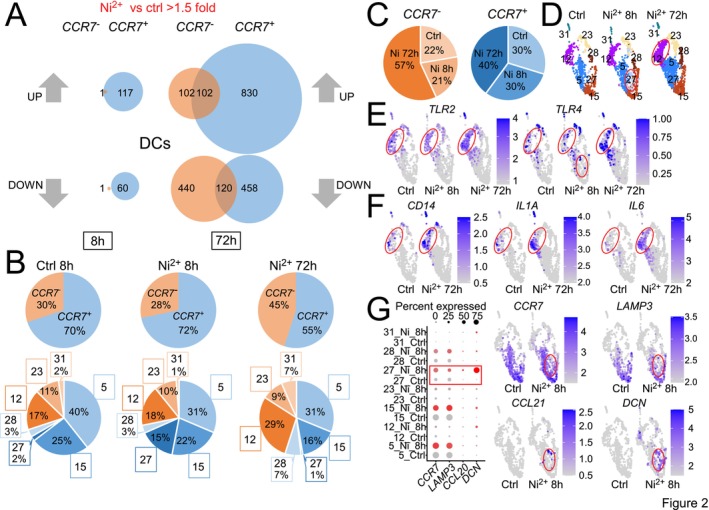
Identification of the Ni^2+^‐responsive DC clusters in human Ni^2+^ allergy. (A) Venn diagrams, showing the numbers of statistically significantly > 1.5‐fold up‐ or downregulated transcripts in the indicated DC subsets after 8 h or 72 h of Ni^2+^ exposure versus the diluent control. (B) Percentage distribution of *CCR7* ^*+*^ (blue) and *CCR7* ^*−*^ DCs (orange) for control and the indicated Ni^2+^‐exposed samples (upper panels) with breakdown of the respective percentages of the individual *CCR7* ^*+*^ (5, 15, 27, 28; blue shadings) and *CCR7* ^*−*^ DC subclusters (12, 23, 31; orange shadings) (bottom panels). (C) Percentage contribution of the designated DC subsets in the specified samples to the total of sequenced *CCR7* ^*+*^ (blue) or *CCR7* ^*−*^(orange) DCs in all three samples. Data were normalized to the determined total cell counts for each treatment. (D) Comparison of the DC compartments cropped out from the individual UMAPs with Ni^2+^‐responsive clusters highlighted in red. (E–G) Excerpts taken from individual UMAPs of the specified samples, showing mRNA expression levels of the indicated genes in single cells (represented as individual dots) within the DC compartment by the intensity of purple shading. Red highlighting marks the positions of the main early and late Ni^2+^‐responsive DC clusters 27 and 12, respectively. A dot plot in (G) additionally shows percentage expression (indicated by the dot size) and relative expression level (illustrated by shading intensity) of the indicated genes in the specified clusters and treatments.

Since DCs play key roles in T cell priming and activation, we characterized the Ni^2+^‐induced transcriptional changes in the DC clusters in detail. After 8 h, only *CCR7* ^*+*^ DCs (clusters 5, 15, 27, and 28) showed significant transcriptional changes, while after 72 h both subgroups contributed (Figure 2A). Relative proportions of *CCR7* ^*−*^ and *CCR7* ^*+*^ DCs within the differently exposed samples showed negligible quantitative changes after 8 h, but we observed a notable increase in *CCR7* ^*−*^ DCs after 72 h of Ni^2+^ stimulation (Figure 2B, upper panels), primarily driven by a roughly twofold increase in cluster 12 (Figure 2B, lower panels). Among the *CCR7* ^*+*^ clusters, cluster 27 accounted for 15% of all DCs in the 8 h Ni^2+^‐exposed sample but was almost absent in control and the 72 h Ni^2+^‐exposed sample. Its appearance correlated with a decreased percentage of cluster 5, and —less prominently—cluster 15, while cluster 28 showed no change (Figure 2B, lower panels). Indeed, pseudotime analysis suggested a trajectory from cluster 5 to cluster 27, indicating that the latter may represent a subset derived from cluster 5 (Figure S7). Consistent with an increased DC fraction in the cellular distribution (Figure S4B, Table S2), we observed elevated DC numbers after 72 h Ni^2+^‐exposure, mainly due to elevated *CCR7* ^*−*^ DC numbers (Figure 2C). Overall, these data suggest an increased proliferation and/or recruitment of distinct DC populations upon Ni^2+^ exposure, and reveal clusters 27 and 12 as the main Ni^2+^‐responsive DC subpopulations in the early and late phases of Ni^2+^‐induced ACD (Figure 2D).

To better characterize the different DC subpopulations, we checked the expression of selected DC subset markers based on recent scRNA‐Seq studies [27]. This identified the largely invariant DC clusters 28 and 23 as classical DC subtypes cDC1 and cDC2, defined by *THBD (CD141)* ^*+*^ *CLEC9A* ^*+*^ *XCR1* ^*+*^or *CD1C* ^*+*^ *ITGAM* ^*+*^, respectively (Figure S8). Cluster 15 contained Langerhans cells, as indicated by the expression of *CD207* (Langerin) and *CD1A*. Together with cluster 5, it further included ‘mature DCs enriched in immunoregulatory molecules’ (mregDCs), characterized by co‐expression of *CCR7* and the maturation marker *LAMP3* (Figure S8) [28]. mregDCs have recently been described as a molecular state acquired by cDC1 and cDC2 cells upon sensing and uptake of antigens [28] and represent cells specialized in antigen presentation. Cluster 12 primarily contained DC3 cells, a *CD5* ^*−*^ *CD163* ^*+*^ *CD14* ^*+*^ cDC2 subpopulation shown to expand in inflammatory diseases [29]. However, clusters 5, 12, and 23 also comprised M1 and M2 macrophages, as indicated by the expression of macrophage markers such as *MRC1* (CD206) and *CD68* (Figure S8).

Considering that Ni^2+^‐dependent CHS induction in mice required human *TLR4* [8] and expression of either *TLR2* or *TLR4* was indispensable for CHS induction by model allergens [30], we examined their presence in the DC subpopulations. *TLR2* expression was high in the late Ni^2+^‐responsive *CCR7* ^*−*^ DC cluster 12 under all stimulation conditions, but absent in the early Ni^2+^‐responsive cluster 27 (Figure 2E). Yet, cluster 27 was the main *CCR7* ^*+*^ cluster besides cluster 5 containing some *TLR4* ^+^ cells (Figure 2E). For cluster 12, we found elevated mRNA levels of *TLR4* and its co‐receptor *CD14* along with induction of the known NFκB target genes *IL6* and *IL1A* in the 72 h Ni^2+^‐exposed sample (Figure 2F). For the Ni^2+^‐specific cluster 27, we did not find typical NFκB‐response genes such as *CXCL2, CCL2 and CCL20* but found *NFKB1* and *REL* as well as prominent NFκB‐dependent maturation genes such as *CD86* and *CD83* among the induced genes. Moreover, cells in this cluster exhibited high expression of MHC class II genes such as HLA‐DRA (Table S3). From their marker expression, they resembled mregDCs supposed to emerge after TLR activation [27] but were typified by a *CCR7* ^*intermediate*^ *LAMP3* ^*low*^ phenotype, some positivity for the CCR7 ligand *CCL21*, and high expression of *DCN* (Figure 2G, Figure S8), an endogenous proteoglycan ligand for TLR2 and TLR4 that can suppress IL10 and enhance expression of the TH1‐driving cytokine IL12 [31, 32].

### 3.4 Ni^2+^ Triggers Early Proinflammatory Gene Expression in ECs and KCs

**FIGURE 3:**
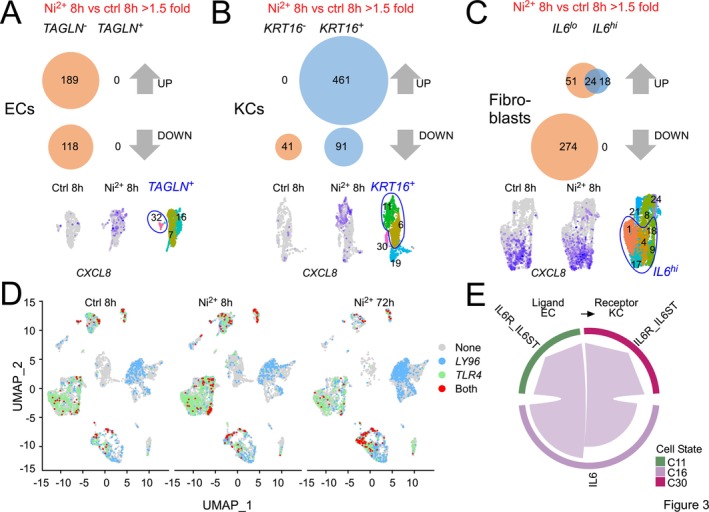
Ni^2+^‐responsiveness of EC and KC subsets and their association with TLR4/LY96 co‐expression. (A‐C) Upper panels: Venn diagrams, showing the number of statistically > 1.5‐fold up‐ or down‐regulated genes in the indicated EC (A), KC (B), or fibroblast subsets (C) after 8 h of Ni^2+^ exposure relative to the diluent‐exposed control. Lower panels: Excerpts from full UMAPs showing mRNA expression levels of the NFκB‐dependent cytokine *CXCL8* [33] in the indicated cell clusters of control and the 8 h Ni^2+^ sample at the single‐cell level as purple shading. (D) UMAP representation of the indicated samples, with single‐cell mRNA expression of *TLR4* (green), *LY96* (blue), or *TLR4/LY96 co‐expression* (red) displayed in binary fashion as either one of two states: as “on‐state” (expression level > 0) represented by the respective coloring of the individual cells, or as “off‐state” (expression level = 0) shown in gray. (E) Interaction analysis of EC and KC clusters in the 8 h Ni^2+^‐exposed samples, showing interactions between source EC clusters and recipient KC cell clusters for the predefined IL‐6 pathway (*p* = 0.02). The chord plot depicts specific ligand–receptor pair interactions between the selected clusters, with arrows indicating the direction of the identified interactions.

We next characterized changes occurring in ECs, KCs, and fibroblasts, the other three early Ni^2+^‐responsive cell types. Comparison of the different cell type‐specific subclusters identified *TAGLN* ^*−*^ EC in cluster 16 and *KRT16* ^*+*^ KCs in cluster 11 as primary subsets showing both relevant DEG upregulation and prominent NFκB target induction such as *CXCL8* (*IL‐8*) after 8 h of Ni^2+^ exposure (Figure 3A,B; Tables S4 and S5). Concerning fibroblasts, the *IL6* ^lo^ subset accounted for most of the Ni^2+−^dependent gene expression, but genes were predominantly suppressed. Clusters 4 and 18 moderately induced *CXCL8*, while the *IL6* ^hi^ subset exhibited basal *CXCL8* expression but no changes upon Ni^2+^ exposure and only minimal DEGs regulation (Figure 3C). Intriguingly, binary analysis of *TLR4* coexpression with its co‐receptor *LY96* (*MD2*), which is indispensable for Ni^2+^‐induced innate immune activation [8] revealed ECs cluster 16 as the only early Ni^2+^‐activated cell subset displaying clear *TLR4/LY96* co‐expression under all conditions (Figure 3C). Suprabasal *KRT16* ^*+*^ KCs, instead, which exhibited the highest number of early Ni^2+^‐regulated DEGs (Figure S5), completely lacked *TLR4* mRNA (Figure 3D). We also found no cellular *TLR4/LY96* co‐expression in the *KRT16* ^*−*^ KC clusters, for which we mainly observed gene suppression upon Ni^2+^‐exposure (Figure 3B). *TLR4/LY96* co‐expression was also not detected in the Ni^2+^‐induced DC cluster 27, but we could confirm their co‐expression in the late Ni^2+^‐responsive DC cluster 10 and cluster 5 (Figure 3D). In cell types with no/minimal early Ni^2+^ responsiveness such as T cells, *TLR4/LY96* co‐expression was typically absent, except for *IL6* ^lo^ fibroblasts that were *TLR4* ^*+*^ *LY96* ^+^, but only weakly Ni^2+^‐responsive.

We next performed CellChat analysis [21] to identify potential receptor‐ligand interactions that may explain the high proinflammatory responsiveness of *TLR4* ^−^ KCs. This revealed IL6 derived from EC cluster 16 as a potential activator of proinflammatory IL6 signaling in KC cluster 11 (Figure 3E). Additionally, the study of possible DC‐T cell interactions indicated an interaction of CCL20 derived from the late Ni^2+^ responsive *CD163* ^*+*^ DC cell subset in cluster 12 with several CCR6^+^ T cell subsets (Figure S9). Consistently, IF staining confirmed increased abundance of CD163^+^ cells in the direct vicinity of infiltrating CD3^+^ T cells 72 h after Ni^2+^ exposure (Figure S10).

Overall, these data highlight EC cluster 16 and DC cluster 12 as relevant *TLR4/LY96*‐positive subsets showing early and late proinflammatory gene expression upon Ni^2+^ exposure.

### 3.5 Massive Infiltration of Central Memory T Cells (T_CM_ ) During Elicitation of Human Ni^2+^ Allergy

**FIGURE 4:**
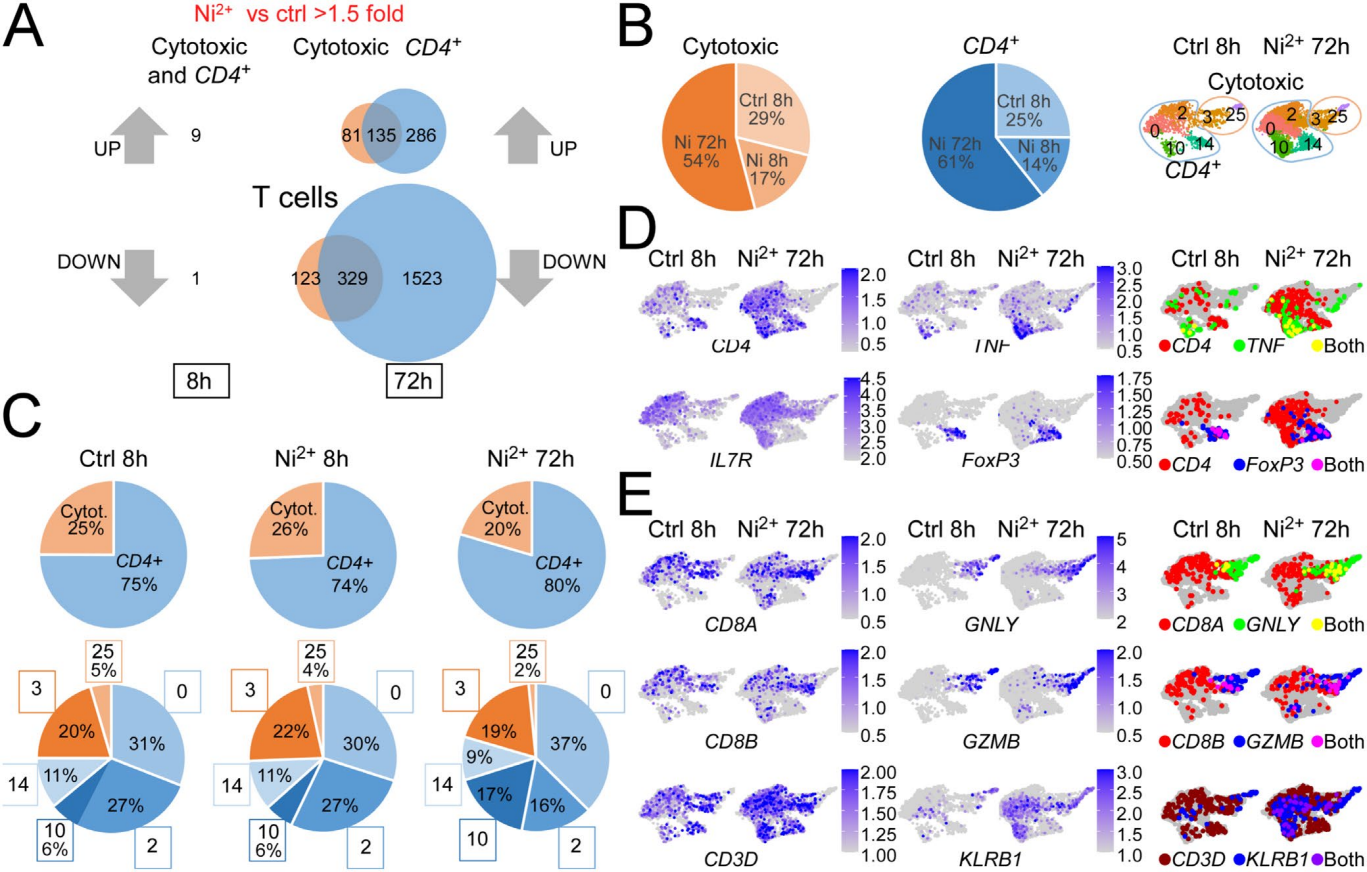
Ni^2+^ fails to trigger an early T cell response but leads to massive recruitment of several T cell subsets during the elicitation phase of human Ni^2+^ allergy. (A) Venn diagrams, showing the number of identified > 1.5‐fold statistically up‐ or downregulated genes for the cytotoxic (orange) or *CD4* ^*+*^ (blue) T cell subsets between the 8 h control and the indicated Ni^2+^‐exposed samples. (B) Total count‐adjusted percentile contribution of the different samples to the total of the two major T cell subsets (*left panels*) and comparison of UMAP sections showing the different T cell subpopulations in the control and the 72 h Ni^2+^‐exposed sample (*right panel*). (C) Relative ratio of CD4^+^ and cytotoxic T cell subsets in the different samples (upper panel) with an additional breakdown of the percentage contribution of the individual *CD4* ^*+*^ (0, 2, 10, 14; blue shading) and cytotoxic T cell clusters (3, 25; orange shading) (*lower panels*). (D, E) mRNA expression level of the indicated T cell markers displayed at single cell level for the respective T cell sections of UMAPs from the diluent‐ (ctrl) or 72 h Ni^2+^‐exposed sample. Purple shading indicates mRNA expression levels of the indicated genes, with scales shown separately for each gene. Additionally, for selected marker genes co‐expressions are shown.

**FIGURE 5:**
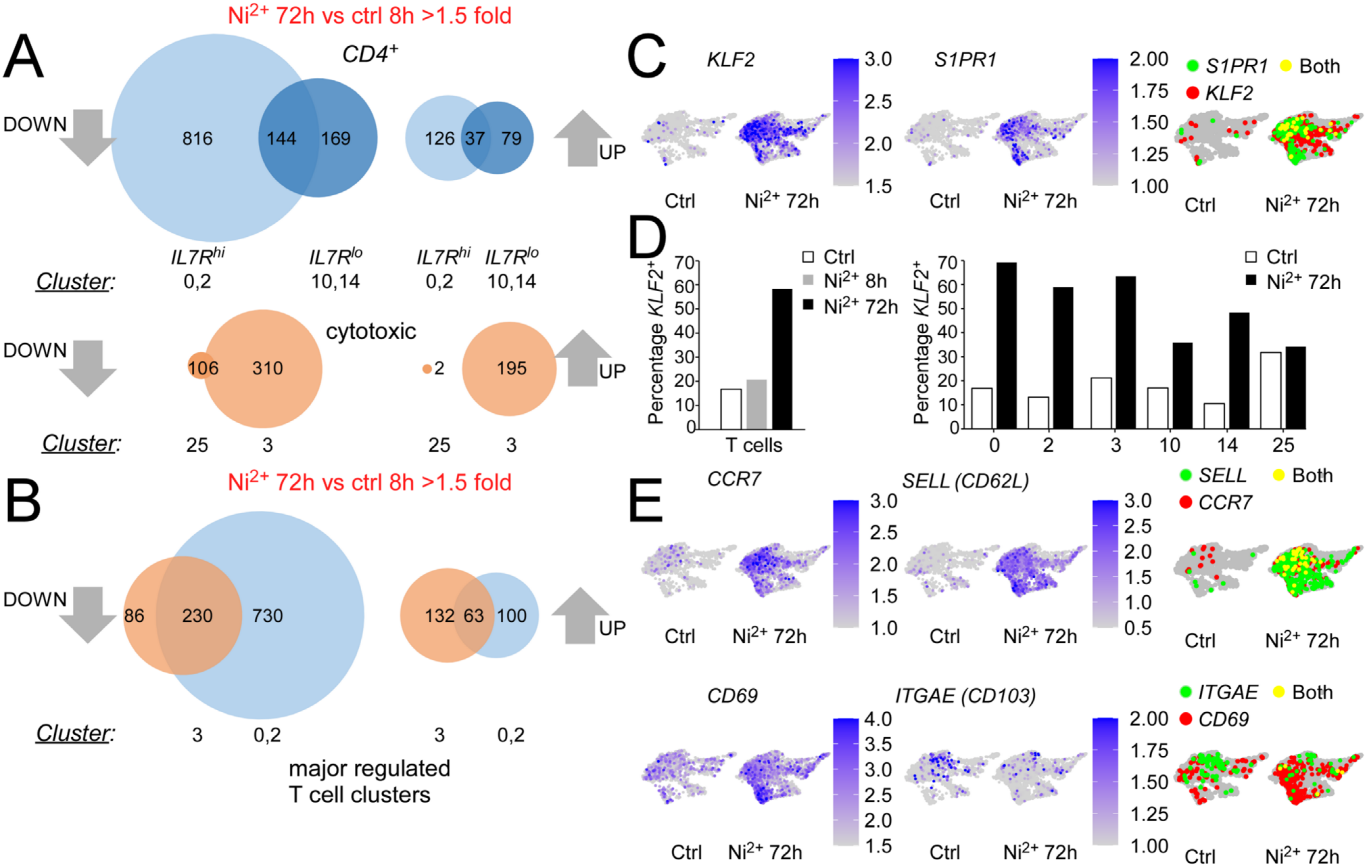
The Ni^2+^‐induced T cell response is dominated by infiltration of *KLF2*‐positive T cells with *CCR7* ^*+*^ */ CD62L* ^*+*^ central memory phenotype. (A) Venn diagrams, showing the numbers of > 1.5 fold statistically Ni^2+^‐regulated DEGs in the indicated memory‐like *IL7R* ^hi^ (light blue) or more differentiated *IL7* ^lo^ *CD4* ^*+*^ (dark blue) subsets (*upper panel*) or cytotoxic T cell subsets (*lower panels*) obtained after cluster‐specific comparison of gene expression between the control and the 72 h Ni^2+^‐exposed samples. (B) Venn diagram, showing a high number of DEGs shared between the *IL7R* ^hi^ *CD4* ^+^ clusters 0 and 2 and the cytotoxic T cell cluster 3. (C) Increased abundance of cells with high mRNA expression of *KLF2* and its target gene *S1PR1* upon 72 h Ni^2+^ exposure. Shown are T cell‐specific UMAP sections of scRNA‐Seq data from the 8 h diluent control and the 72 h Ni^2+^‐stimulated samples, with mRNA expression of the respective genes displayed at single cell level by purple shading. (**D**) Bar charts, showing percentages of *KLF2* ^+^ cells within the total T cell subset for the indicated treatments (*left*) or the specified individual T cell clusters in the 8 h diluent controls (Ctrl) or the 72 h Ni^2+^‐exposed samples (*right*). (E) T cell‐specific sections of sample‐specific UMAPs similar to those in (**C**), but showing individual or combined expression of the T_CM_ markers *CCR7* and *SELL* or the T_RM_ markers *CD69* and *ITGAE* (*CD103*), respectively.

T_RM_ have been implicated as key players in CHS responses to model allergens and Ni^2+^ in mice [15]. We thus examined the exact contribution of the identified T cell populations in the early and late phases of human Ni^2+^ allergy. Comparing gene expression of *CD4* ^*+*^ and cytotoxic T cell subsets separately between control and the Ni^2+^‐exposed samples, we found minor DEG expression after 8 h of Ni^2+^ exposure but detected overall 502 upregulated and 1975 downregulated genes after 72 h (Figure 4A). Remarkably, *CD4* ^*+*^ and cytotoxic T cells shared a substantial number of DEGs and there was no clear preference in Ni^2+^ responsiveness between the two main T cell subclusters, albeit overall more DEGs were detected for the *CD4* ^*+*^ subset (Figure 4A). Analyzing total T cell counts, we noticed a slight drop for both subsets early after Ni^2+^ exposure (Figure 4B), but this reduction was nonspecific and similar to that seen upon 8 h Cr_2_O_7_^2−^ exposure (Figure S4B). After 72 h Ni^2+^ exposure, however, cell numbers for both subsets were increased (Figure 4B). The ratio of both subsets in the different samples remained largely constant, albeit the balance slightly shifted towards CD4^+^ T cells after 72 h (Figure 4C). Thus, the overall increase in total T cell numbers apparently resulted from a global recruitment of Ni^2+^‐specific T cells and not recruitment/proliferation of a specific T cell subset. Analysis of additional T cell lineage markers confirmed an overall increase of CD4^+^ cells defining the TH cell populations in clusters 0, 2, 10, and 14 (Figure 4D, Figure S11A). These clusters included the *FOXP3* ^*+*^ Treg and *TNF* ^*+*^ TH1 subpopulations present in clusters 14 and 10 (Figure S11A), of which the latter was clearly increased (Figure 4D). Other TH responses such as TH17 polarization were not clearly evident as the percentage expression of typical TH17 markers such as *RORC*, *IL17A*, *IL17F*, *IL23A*, or *IL23R* was overall low and mainly found in the predominant TH1 cluster 10 (Figure S11B). Also, re‐clustering of the T cells did not change this (data not shown). Besides TH1 cells, we also observed elevated numbers of *CD8A* ^*+*^ *GNLY* ^*+*^ *GZMB* ^*+*^ *KLRB1* ^*+*^ cytotoxic T cells in cluster 3 but not of *CD3* ^*−*^ *KLRB1* ^*+*^ natural killer (NK) cells in cluster 25 (Figure 4E, Figure S11A). Statistical comparison of the 72 h Ni^2+^‐exposed sample and the control validated significantly increased cell numbers in all individual T cell clusters but cluster 25 (Table S6). Comparative analysis of Ni^2+^‐induced gene expression for individual cytotoxic T cell subsets, the memory‐like *IL7R* ^hi^ CD4^+^ T cell clusters 0 and 2, and the more differentiated *IL7R* ^lo^ CD4^+^ clusters 10 and 14 (Figure 4D) revealed the highest number of Ni^2+^‐regulated DEGs in the *CD4* ^*+*^ cluster 0, followed by the cytotoxic T cell cluster 3 (Figure 5A). An appreciable number of Ni^2+^‐regulated genes was also found for the *IL7R* ^lo^ clusters 10 and 14 comprising the TH1‐polarized *CD4* ^*+*^ cells and Treg cells, but almost no DEGs were found for the NK cluster 25 (Figure 5A). A comparison of Ni^2+^‐regulated transcripts between cluster 3 and the *IL7R* ^hi^ CD4^+^ clusters 0 and 2 revealed a substantial overlap of DEGs (Figure 5B, Table S7). Among the top shared Ni^2+^‐upregulated transcripts was *KLF2* (Figure 5C,D; Table  S7), an essential transcription factor regulating T cell trafficking to peripheral tissues by controlling expression of *S1PR1*, *SELL (L‐Selectin, CD62L)* and *CCR7* [34, 35, 36]. Its expression confers survival of memory T cells [37], whereas T_RM_ lack expression of *KLF2* and its target *S1PR1* [38]. Similar to *KLF2*, cellular *S1PR1* expression was elevated in the T cell subsets of the 72 h Ni^2+^‐exposed sample, but was more restricted and mainly evident in the *IL7R* ^hi^ CD4^+^ T cell clusters 0 (Figure 5C). Moreover, we observed strongly increased numbers of *CCR7* ^*+*^ *SELL* ^*+*^ T cells in cluster 0 (Figure 5E, Figure S11C), which mark T_CM_ [39, 40]. By contrast, we found fewer cells expressing *ITGAE (CD103)*, which together with *CD69* characterizes T_RM_ [39, 40]. Cellular *CD69* was slightly higher in all T cell clusters, particularly cluster 10 (Figure 5E), but as *CD69* is classically considered as an early marker of T cell activation [41] this likely reflected enhanced T cell activation in the Ni^2+^ 72 h sample.

**FIGURE 6:**
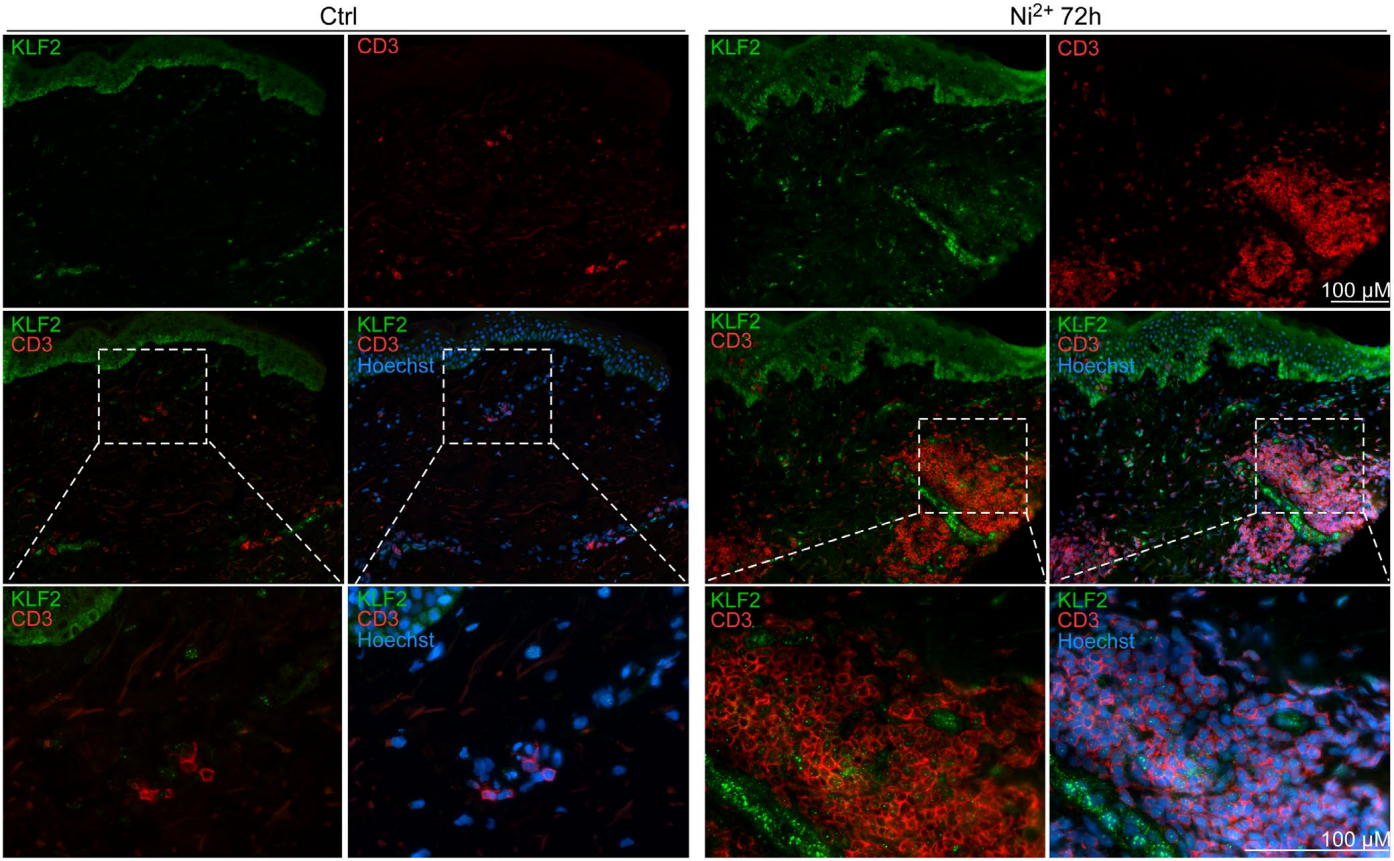
Epicutaneous exposure of sensitized donors with Ni^2+^ results in massive skin infiltration of KLF2^+^ T cells. Indirect immunofluorescence staining of cryosections of diluent‐ (ctrl) or 72 h Ni^2+^‐exposed donor skin, in which the presence of T cells with positive nuclear KLF2 staining was identified by co‐staining with a monoclonal antibody for CD3 (as a pan‐T cell marker, red) and a KLF2‐specific antiserum (green), followed by antibody detection using appropriate fluorescence‐coupled secondary antibodies. Additional Hoechst staining identified cell nuclei. Both single‐channel images (top) as well as overlay images of the indicated stainings recorded at 200× magnification are shown. Nuclear KLF2 positivity in T cells manifested as bright green dot‐like structures within the nucleus of red‐labeled CD3^+^ cells. Lower panels show cropped higher magnifications of the indicated areas from the overlay images in the middle (white dotted areas) recorded at 400× magnification. Data were reproduced with similar results using cryofixed tissue from three independent donors.

To investigate whether the observed global recruitment of *KLF2* ^*+*^ T cells required sensitization and was Ni^2+^‐specific, we performed additional immunofluorescence staining. Investigation of independent Ni^2+^‐sensitized patients confirmed a massive skin infiltration of KLF2^+^ CD3^+^ T cells upon 72 h epicutaneous Ni^2+^ exposure, especially near blood vessels in the dermis, indicating that KLF2 positivity was particularly high among freshly infiltrating T cells (Figure 6). By contrast, very few CD3^+^ T cells were detected in the dermis upon diluent treatment, and these cells were predominantly KLF2^−^ (Figure 6). Ni^2+^ exposure of a budesonide‐allergic patient not sensitized for Ni^2+^ failed to induce KLF2^+^ T cell infiltration, but budesonide challenge also triggered massive KLF2^+^ T cell recruitment (Figure S12), implying that their infiltration requires sensitization but is independent of the hapten structure.

## 4 Discussion

This study provides the first comprehensive single‐cell transcriptome analysis of human Ni^2+^ allergy, identifying three key cell types in the early transcriptional response. First, *TAGLN* ^*−*^ *LYVE* ^*−*^ *TLR4* ^*+*^ *LY96* ^*+*^ ECs emerged as early proinflammatory responders, confirming ECs as one of the first cells activated in Ni^2+^‐induced ACD [42]. Second, we identified early proinflammatory activation of *TLR4* ^*−*^ *KRT16* ^*+*^ KCs, likely as a secondary consequence of cytokine release such as IL6 from *TLR4* ^*+*^ *LY96* ^*+*^ cells, as indicated by our CellChat analysis, since both *TLR4* ^−^ KCs and epidermal skin were insensitive for Ni^2+^‐induced activation [7, 11]. This implies a role as an amplifier of inflammation rather than an initiator, consistent with KCs inhibiting T cell effector function under steady‐state conditions [12]. It further highlights a function of *TAGLN* ^*−*^ ECs as initiators of the early inflammatory response, as they were the major native *TLR4* ^*+*^ *LY96* ^*+*^ cells showing considerable proinflammatory responsiveness early upon Ni^2+^ exposure. We cannot exclude, however, that certain short‐lived cells, such as neutrophils implicated in Ni^2+^‐induced ACD [43] may contribute, as they escaped our scRNA‐Seq analysis due to our isolation strategy. Notably, *TLR4*/*LY96* co‐expression alone was insufficient to predict proinflammatory Ni^2+^ reactivity and cellular localization may equally be relevant. For instance, fibroblasts were *TLR4* ^*+*^ *LY96* ^*+*^ and Ni^2+^ responsive in vitro [11] but characterized by only moderate early inflammatory activation upon Ni^2+^ exposure (*IL6* ^lo^ subset) or no change over basal proinflammatory gene expression (*IL6* ^hi^ subset).

Among the Ni^2+^‐regulated DC populations, we identified two putatively relevant subsets: First, the *CCR7* ^*+*^ *DCN* ^*+*^ cluster 27 that resembled mregDCs, but showed lower expression of the maturation marker *LAMP3*. This cluster emerged as an early but transient Ni^2+^‐specific DC subpopulation and may represent a subset of tolerogenic mregDCs lost during Ni^2+^‐induced ACD, or signify a transient differentiation state of activated DCs later relocalizing to the dermal lymph node as implied by the high expression of HLA antigens and inflammation‐relevant genes. Its appearance coincided with a decrease of *TLR4* ^*+*^ *LY96* ^*+*^ mregDC cluster 5, suggesting Ni^2+^could trigger TLR4‐dependent differentiation of tolerogenic mregDCs into a T cell‐activating subset, a view consistent with our pseudotime analysis, suggesting cluster 5 as a potential precursor of cluster 27. Notably, *DCN* has recently been implicated as a binding partner of TLR4, suppressing IL10 expression and promoting IL12‐dependent TH1 responses [31, 32]. Hence, cluster 27 may contribute to the observed TH1 response in human nickel allergy.

A second possibly relevant DC subpopulation is the late Ni^2+^‐activated *ITGAM* ^*+*^ *CCR7* ^*−*^ DC cluster 12. This cluster mainly contained DC3 cells, an inflammatory *CD163* ^*+*^ *CD14* ^*+*^ *CD5* ^*−*^ cDC2 subpopulation supposed to promote TH17 polarisation [29]. Consistently, we found a statistically significant interaction of cluster 12‐derived CCL20 with CCR6 expressed in T cell cluster 2. Interestingly, CCR6 was reported to define a subset of activated memory T cells of TH17 potential in immune thrombocytopenia [44]. Thus, cluster 12 may contribute to Ni^2+^‐induced TH17 differentiation supposed to be relevant in human Ni^2+^ allergy [45], although we failed to detect a clear Th17 signature in our study. However, TH17 marker genes are notoriously hard to detect in scRNA‐Seq studies [46] and we might have missed a TH17 response due to the chosen timing of sample generation.

Regarding the T cell compartment, we found no evidence for a relevant contribution of T_RM_ in the early phase after a single challenge with Ni^2+^ in sensitized individuals. Secondly, we observed increased infiltration of KLF2^+^ T cells after 72 h of Ni^2+^ exposure. KLF2 expression marks quiescent, migratory T cells critical for T cell homeostasis [37, 47] but is absent in T_RM_ [38]. KLF2 further regulates the expression of the established T_CM_ markers *S1PR1*, *CCR7*, and *SELL* [34, 35, 36] that we equally found upregulated in the T cell compartment after 72 h of Ni^2+^ exposure and has recently been reported to maintain lineage fidelity of effector T cells and prevent T cell exhaustion by suppressing the transcription factor TOX [48]. Thus, the Ni^2+^‐induced T cell response appears to be mainly driven by T_CM_ infiltration, a view well compatible with the typically delayed patch test responses of sensitized patients. However, T_CM_ are also effective T_RM_ precursors in human skin [49]. T_RM_, therefore, might develop from T_CM_ following repetitive or sustained hapten challenges. We did not find increased frequencies of *CD103* ^*+*^ *CD69* ^+^ T_RM_ at early time points up to 72 h after a single Ni^2+^ exposure. However, we did not perform repetitive Ni^2+^ challenges and cannot exclude that the establishment of T_RM_‐based immunity may occur later than 72 h after Ni^2+^ contact. Still, considering that KLF2^+^ T cell recruitment was reproduced upon budesonide‐induced ACD, T_CM_ recruitment and differentiation into effector T cells is likely the default adaptive response to chemically distinct haptens in sensitized patients.

## Author Contributions

Conceptualization: M.S., M.G., and P.K. Investigation: A.K., K.M., and F.I. Validation: K.M. Visualization: M.S., P.K., Y.R., and S.G. Resources: M.G. and S.G. Funding acquisition: M.S. and M.G. Project administration: M.S. and F.I.; Formal analysis: P.K., M.S., and Y.R. Writing‐original draft: M.S. and P.K. Writing‐review and editing: M.S., M.G., Y.R., and P.K.

## Conflicts of Interest

The authors declare no conflicts of interest.

## Supporting information


**Table S1:** all70108‐sup‐0001‐TableS1.xlsx.

**Table S2:** all70108‐sup‐0002‐TableS2.xlsx.

**Table S3:** all70108‐sup‐0003‐TableS3.xlsx.

**Table S4:** all70108‐sup‐0004‐TableS4.xlsx.

**Table S5:** all70108‐sup‐0005‐TableS5.xlsx.

**Table S6:** all70108‐sup‐0006‐TableS6.xlsx.

**Table S7:** all70108‐sup‐0007‐TableS7.xlsx.

**Appendix S1:** all70108‐sup‐0008‐AppendixS1.pdf.

## Data Availability

All raw and processed sequencing data are available at the NCBI Gene Expression Omnibus (GEO; https://www.ncbi.nlm.nih.gov/geo/) under accession number GSE245577.
