# Supplementary material for: Single Cell Analysis Reveals Dynamic Changes of Distinct Cell Populations in Human Nickel Allergy
Source: Allergy. 2025 Oct 15;81(4):1138–48. doi: 10.1111/all.70108 (PMC13040661; doi:10.1111/all.70108)
Supplement: Supplementary file 8 — Appendix S1: all70108‐sup‐0008‐AppendixS1.pdf. [file ALL-81-1138-s002.pdf]

Supplementary Information

Single cell analysis reveals dynamic changes of distinct cell populations in human nickel allergy

Marc Schmidt[1,3], Andrea Knorz[1], Katharina Meder[1], Simon Goller[1], Fabian Imdahl[2], Yamila Rocca[1], Matthias Goebeler[1], and Pierre Khoueiry[2]

[1] Department of Dermatology, University Hospital Würzburg, Würzburg, Germany

[2] Single Cell Center Würzburg, Helmholtz Institute for RNA-based Infection Research (HIRI), Würzburg, Germany

[3] Correspondence to: Marc Schmidt, Department of Dermatology, Venereology and Allergology, University Hospital Würzburg, Josef-Schneider-Str. 2, 97080 Würzburg, Germany. Phone: +49 931 201 26396, Fax: +49 931 201 26462, Email: Schmidt_M11@ukw.de

Contents

Supplementary Materials and Methods 2

Sample processing and single cell isolation 2

Cell labelling, library generation and scRNA-Seq 2

Receptor-ligand interaction and Pseudotime analysis........ 2

.Immunofluorescence Analysis........ 3

Supplementary Figure legends........ 4

Supplementary Table Legends 8

Supplementary References 10

Supplementary Figures........ 11

Supplementary Materials and Methods

Sample processing and single cell isolation

Punch biopsies were rinsed once in 70% ethanol and twice in sterile PBS, freed of fat, and cut into small pieces. Pieces were incubated overnight at 37°C in RPMI medium containing 10% FCS, 1% penicillin/streptomycin, 8 µg/ml collagenase type IV and 20.1 µg/ml DNAse I and cells released using a gentleMACS dissociator (Miltenyi). Isolated single cells were separated from undissociated tissue by filtering through a 100-µm cell strainer and dead cells removed using a commercial kit (Miltenyi). Subsequently, concentrations of the single cell suspensions were adjusted appropriately for labelling and scRNA-Seq.

Cell labelling, library generation and scRNA-Seq

For single-cell gene expression analysis, cells were partially multiplexed using TotalSeq Hashtag antibodies or the 10x Genomics 3' CellPlex Kit. Following, single-cell suspensions were loaded onto the 10x Genomics Chromium Controller to generate single-cell Gel Bead-Emulsions (GEMs). Within these GEMs, individual cells were lysed, and unique barcoded cDNA was reverse transcribed from mRNA. Subsequently, libraries were generated using the Chromium Next GEM Single Cell 3' Reagent Kit v.3.1 or the GEM-X 3' Reagents, following the manufacturer's instructions. Library preparation involved several steps, including GEM-RT cleanup and cDNA amplification, fragmentation, adaptor ligation, and sample indexing, to prepare the DNA for Illumina based sequencing. Single-cell libraries were sequenced on an Illumina NovaSeq platform.

Receptor-ligand interaction and Pseudotime analysis

To infer cell–cell communication between different cell subsets, we employed the CellChat algorithm (http://www.cellchat.org/) within the R environment. CellChat quantitatively infers and analyzes intercellular communication networks from single-cell RNA-seq data by leveraging gene expression information of ligand–receptor pairs, based on a curated human pathway database as a reference [1].

From the Seurat object, communication probabilities were calculated for each ligand–receptor interaction.

For visualization, source and target clusters were manually defined alongside unbiased global views. We generated bar plots displaying the top 30 signaling pathways ranked by interaction strength. The netVisual_chord_gene function was used to visualize specific ligand–receptor pairs within each pathway, illustrating directional connections from source to target clusters. The netVisual_aggregate function was employed to produce circle plots summarizing overall communication from selected clusters to others without specifying particular ligand–receptor pairs. Finally, netVisual_bubble was applied to depict communication probabilities of specific ligand–receptor pairs within a given pathway.

Trajectory analysis of scRNA-seq data was performed using the pseudotime algorithm [2] implemented in the Monocle3 package in the R environment (https://cole-trapnell-lab.github.io/monocle-release/). This algorithm leverages gene expression data to construct a single-cell trajectory that represents a dynamic biological process, ordering cells from less activated/undifferentiated states to more activated/differentiated states.

For this analysis, the starting cell population for the DC compartment was defined as the undifferentiated CCR7$^+$ dendritic cell (DC) cluster. Cells were ordered along the inferred trajectory based on similarities in their gene expression profiles. Genes whose expression was significantly correlated with pseudotime were then identified. Finally, pseudotime was visualized as a continuous variable projected onto the UMAP embedding, using a color gradient to reflect progression along the trajectory.

.Immunofluorescence Analysis

6-µm cryosections from three Ni^{2+} ^{pos}, $Cr_2O_7^{2-}$ ^{neg} donors exposed to vaseline (control) or 5% $NiSO_4$ or from a donor sensitized to budesonide but not Ni^{2+} and exposed to vaseline, 5% $NiSO_4$, or 0.1% budesonide for 72h, were fixed in a 1:2 acetone/methanol mixture for 10 min at -20°C. After two

rinses with PBS, samples were blocked in 2% BSA/PBS with 10% donkey serum (Linaris END9010-2) (30 min, RT) and co-stained overnight at 4°C with a mixture of a rabbit αKLF2 antibody (Sigma HPA 055964) diluted 1:100, or a mouse αCD163 antibody (Leica Biosystems NCL-L-CD163) diluted 1:500,together with a 1:300 dilution of rat αCD3 antibody (clone CD3-12, BioRad MCA 1477T) in antibody dilution buffer (2% BSA/PBS with 5% donkey serum), respectively. Samples were washed three times with PBS containing 2% BSA/0.1% Triton X and $CD3^+$ $KLF2^+$cells or $CD3^+$ $CD163^+$ cells detected by 45 min incubation with a mixture of Cy3-coupled α-rat and either a Cy5-coupled α-rabbit secondary antibody or an α-mouse Dy647 antibody from donkey (Jackson, each diluted 1:100 in antibody dilution buffer). After removal of excess secondary antibodies and staining of nuclei with Hoechst 33342 (Sigma, 1:1000 in PBS), sections were mounted onto a glass slide in appropriate embedding medium (IBIDI) and analysed at 200x or400x magnification by multicolour fluorescence microscopy using a Nikon Ti fluorescence microscope.

Supplementary Figure legends

Figure S1 Overview of the design of the scRNA-Seq study. (**A**) Schematic scheme of the timelines and samples included in the scRNA-Seq. Samples of three independent donors sensitized for Ni^{2+} but insensitive for the unrelated metal allergen $Cr_2O_7^{2-}$ were included. All three donors epicutaneously received vaseline (V) for 8h as negative control and 5% $NiSO_4$ (Ni^{2+}) for 8h and 72h. One of the donors additionally was exposed to 0.5% $K_2Cr_2O_7$ ($Cr_2O_7^{2-}$) for 8 and 72h. To control batch effects and confirm reactivity, 72h samples were stimulated 64h before the 8h samples and 5mm (8h time points) or 8mm punch biopsies (72h time points) taken simultaneously. In all cases, single cells were isolated freshly and processed for multiplexed scRNA-Seq, as detailed in the Supplementary Material and Methods. (**B**) Scheme of the localization of the epicutaneous exposure site with distances of the respective exposures. (**C**) Representative patch test reactions at the time point of biopsy, 72h after Ni application for the late stimulation time point.

Figure S2 Reliability of the performed cell annotation. Violin diagrams, showing the linear distribution of mRNA expression of selected cell type-specific markers in the indicated annotated cell types as confirmation of the reliability of the performed cell typing.

Figure S3 Interrelation and ancestry of the different identified cell clusters in human Ni^{2+} allergy. Clustering tree plot, showing the relationship between the different identified cell clusters in the combined scRNA-Seq data set of Ni^{2+}-, $Cr_2O_7^{2-}$- or diluent-exposed donor cells from the included three Ni^{2+}-sensitized patients at different resolution. Red highlighting shows the finally chosen resolution for generating the UMAP representation in Fig. 1A, with cluster numbers at this resolution corresponding to those in Fig. 1A.

Figure S4 Ni^{2+}-dependent changes in cell distribution during the course of human Ni^{2+} allergy. (**A**) Sample-specific UMAP representation of the scRNA-Seq data set from Fig. 1A, confirming the absence of the Ni^{2+}-specific changes shown in Fig. 1C for the $Cr_2O_7^{2-}$-stimulated negative controls. (**B**) Barplot, showing mean changes in the percentage distribution of the indicated skin cell types upon epicutaneous exposure of the three included $Ni^{2+\,Pos}$ $Cr_2O_7^{2-\,Neg}$ donors with Ni^{2+} or $Cr_2O_7^{2-}$ for 8 or 72h, respectively.

Figure S5 ECs, KCs, DCs and fibroblasts are the major Ni^{2+}-responsive cells in the early phase of human Ni^{2+} allergy. (**A**) Barplot, showing the total numbers of DEGs in the annotated cell types between the integrated three 8h Ni^{2+}-exposed samples and the respective 8h diluent controls. Statistically upregulated and downregulated genes are shown in a stacked bar representation in black and grey colour with values above the bars indicating the total number of >1.5 fold significantly ($p\_adj < 0.05$) regulated DEGs. (**B**) Stacked bar chart as in (**A**), but with the total number of early Ni^{2+}-regulated DEGs broken down for the indicated cell subpopulations.

Figure S6 Subclustering of the identified main cell clusters by discriminatory marker expression. (**A-E**) Violin diagrams, showing the expression of the indicated discriminatory markers for the identified DC clusters (**A**), EC clusters (**B**), KC clusters (**C**), T cell subpopulations (**D**) and fibroblast subsets (**E**). Expression levels of the indicated genes in cells of the specified UMAP clusters are shown as linear

distribution of normalized mRNA expression values, with the expression level of the respective selected main discriminatory marker in individual cells displayed as single black dots.

Fig. S7 Pseudotime analysis suggests cluster 5 as potential origin of the Ni^{2+}-specific cluster 27. (**A**) UMAP representation of skin CCR7⁺ DC clusters identified by scRNA-seq, showing the inferred transcriptional trajectory connecting the selected clusters (5, 15, 27, 28) as determined by pseudotime analysis. The Monocle-derived trajectory is shown, starting from the selected, less-differentiated root (cluster 5) and progressing through the different clusters along the pseudotime scale, colored from blue to yellow. (**B**) Barplot showing re-ordered CCR7⁺ DC clusters identified in Seurat, plotted against increasing median pseudotime, as calculated by Monocle, indicating the inferred directionality of DC differentiation and/or activation. (**C**) Pseudotime analysis of selected genes (CD207, CSF2, CXCL2, CXCL3, CXCL8, FCER1G, IKZF2, IL1B, SELL, SLAMF9, TLR2, TLR4) that significantly contribute to the pseudotime trajectory. The x-axis represents pseudotime, and the y-axis represents gene expression levels.

Figure S8 Classification of the identified DC subclusters according to their signature marker expression. (**A**) Dotplot, showing expression of the indicated DC marker genes, defining distinct DC subsets in the indicated cell clusters. Key markers for the specified subset are highlighted by black of blue colour. Dot sizes correspond to the percentages of expression with colour intensities indicating expression levels. (**B**) Excerpts from full UMAPs of the integrated scRNA-Seq dataset showing mRNA expression of individual signature marker genes at single cell level by purple shading or co-expression of relevant markers for classical DC1 (cDC1; *THBD*, *CLEC9A*, *XCR1*), cDC2 (*CD1C*, *ITGAM*), Langerhans cells (*CD207*, *CD1A*), cDC3 (defined as *CD163*$^{+}$, *CD14*$^{+}$, *CD5*$^{-}$) and mregDC (*CCR7, LAMP3*) in the identified DC clusters in yellow. In addition, mRNA expression of the macrophage (MP) markers *MRC1* and *CD68* and the pan-DC marker *ITGAX* are displayed. For reference, an excerpt of the UMAP from Fig. 1A with an overview of the individual identified DC subclusters is shown in the upper right corner.

Figure S9 Interaction of DC3-derived CCL20 with different CCR6 expressing T cell subsets. (**A**) Interaction analysis of DC and T cell clusters in the 72 h Ni^{2+}-exposed samples, showing interactions between source DC clusters and recipient T cell clusters for the predefined CCL pathway (p-value < 0.001). The chord plot depicts specific ligand–receptor pair interactions between the selected clusters, with arrows indicating the direction of the identified interactions. (**B**) Dot plot, showing expression of CCR6 and CCL20 in the indicated DV and T cell clusters. Dot sizes indicate percentages of expression with levels of expression indicated by colour shading from yellow (low) to purple (high).

Fig.S10 Increased presence and co-localization of $CD163^+$ cells in the vicinity of infiltrating $CD3^+$ T cells during the elicitation phase of human nickel allergy. Indirect Immunofluorescence co-staining of CD163 and CD3 performed on cryosections from a Ni^{2+}-sensitized donor epicutaneously exposed to Ni^{2+} for 72h. $CD3^+$ T cells and $CD163^+$ putatively representing DC3 cells were identified using combinations of specific primary and appropriate fluorescence-coupled secondary antibodies. Data show enhanced presence of $CD163^+$ cells (red) partially in close vicinity of $CD3^+$ infiltrating T cells (green) after 72h of Ni^{2+} exposure. Shown are single channel images (top) as well as two- or three-colour overlays of the specified stainings recorded at 200x magnification (middle) as well as cropped magnifications of the indicated areas in the overlay images in the middle recorded at 400x magnification (bottom). Data are representative of similar stainings of cryosections obtained from n=3 independent donors.

Fig. S11 Characterization of the identified T cell clusters on basis of signature marker expression. Dotplot representation, showing expression and abundance of selected T cell signature markers characterizing main T cell identities ($CD4^+$ Treg: *CD4*, *FOXP3*, TH1: *CD4*, *TNF*, cytotoxic $CD8^+$ T cells: *CD8A*, *CD8B*, *CD3D*, *GNLY*, *GZMB* and *KLRB1*, NK cells: *GNLY*, *GZMB*, *KLRB1*, lack of *CD3D*) (**A**), main TH subsets (TH1: *TBX21*, *TNF* and *IFNG*; TH2: *GATA3* and *IL4*, TH17: *RORC*, *IL17A*, *IL17F*, *IL23R* and *IL23*) (**B**) and specific memory T cell populations (T_{CM}: KLF2, CCR7, SELL; T_{RM}: *ITGAE*, *CD69* (**C**), respectively. In (**A**) and (**C**) additionally *MKI67* and *TOX* expression were assessed to grossly evaluate the degree of proliferation and exhaustion in the individual T cell clusters, respectively. Dot sizes

indicate percentages of expression with levels of expression indicated by colour shading from yellow (low) to purple (high).

Figure S12 Contact allergen-induced infiltration of KLF2$^+$ T cells at the elicitation phase occurs independently of the sensitizing allergen. Indirect Immunofluorescence co-staining of KLF2 and CD3 performed on cryosections from a budesonide- but not Ni^{2+-}sensitized donor epicutaneously exposed to Ni^{2+} or budesonide for 72h. T cells with nuclear KLF2 expression were identified using combinations of specific primary and appropriate fluorescence-coupled secondary antibodies. Green dot-like staining in the nucleus of red-labelled CD3$^+$ cells indicates presence of KLF2$^+$ T cells. Shown are single channel images (top) as well as two- or three-colour overlays of the specified stainings recorded at 200x magnification (middle) as well as cropped magnifications of the indicated areas in the overlay images in the middle recorded at 400x magnification (bottom).

Supplementary Table Legends

Supplementary Table 1. Overview of the total cell counts included in the presented scRNA-Seq analysis. Table listing total cell counts obtained for the respective single clusters of the identified cell types shown separately for the respective treatments. Annotated main cell types are indicated by identical colour. Total counts are derived from n=3 patients sequenced separately and integrated into one single analysis.

Supplementary Table 2. Statistical evaluation of changes in cell type distribution. Statistical evaluation of altered cell counts in the indicated Ni^{2+} treatments compared to the experimental vehicle control (ctrl) or between the early (Ni8h) and late Ni^{2+}-exposed (Ni72h) time points as determined by chi-squared test followed by bonferroni correction. Adjusted p-values of <0.05 were considered significant.

Supplementary Table 3. Marker gene expression, characterizing the early Ni^{2+}- responsive DC cluster 27. Table listing genes statistically (p.adj < 0.05) >1.5 fold up- (red) or downregulated (green) by average (avg_FC) in the early N^{i2+}-responsive cluster 27 in relation to all other cells in the

sequenced dataset. White highlighting indicates MHC class II genes, yellow highlighting denotes genes encoding known NFκB responsive maturation genes such as CD86 or regulated components of the NFκB pathway such as *REL.* Pct.1 signifies percentage expression in cluster 27, pct.2 percentage expression in all other cells, respectively.

Supplementary Table 4. List of early Ni^{2+}-regulated DEGs in $TAGLN^-$ ECs. Table showing statistically (p.adj. <0.05) > 1.5 fold up- (red) or downregulated genes (green) found upon comparison of gene expression of the *$TAGLN^-$* fraction EC fraction present in the 8h Ni^{2+}-exposed treatments versus the 8h vehicle-exposed control. Yellow highlighting marks induced genes previously found to be Nickel-regulated in an NFκB-dependent manner in human primary ECs [3]. Pct.1 signifies percentage expression of the respective genes in the $TAGLN^-$ EC subset of the 8h Ni^{2+}-exposed samples, pct.2 the percentage expression in the vehicle control, respectively.

Supplementary Table 5. List of early Ni^{2+}-regulated DEGs in $KRT16^+$ KCs. Table showing statistically (p.adj. <0.05) > 1.5 fold up- (red) or downregulated genes (green) found upon comparison of gene expression of the $KRT16^+$ KC fraction present in the 8h Ni^{2+}-exposed treatments versus the 8h vehicle-exposed control. Yellow highlighting marks established proinflammatory NFκB-dependent Nickel target genes [3]. Pct.1 signifies percentage expression of the respective genes in the $KRT16^+$ KC subset of the 8h Ni^{2+}-exposed samples, pct.2 the percentage expression in the vehicle control, respectively.

Supplementary Table 6. List of T cell clusters statistically induced upon 72h Ni^{2+} exposure. Shown are total cell counts of the indicated T cell cluster in the 8h vehicle control (Ctrl_8h) (n.sample1) and the corresponding 72h Ni^{2+}-exposed sample (Ni_72h) (n.sample 2) as well as the respective total cell counts not belonging to the respective T cell cluster for the corresponding treatments(not.sample 1, not.sample2), respectively. Statistical differences between the two treatment groups were evaluated by chi-squared test followed by bonferroni adjustment and adjusted p-values (p.adj) <0.05 considered significant.

Supplementary Table 7 List of uniquely and commonly up- or downregulated genes in the memory like CD4$^+$ IL7R^{hi} T cell subset and CD8$^+$ cytotoxic T cells. Tables show statistically (p.adj<0.05) >1.5 fold up- (red) or downregulated (green) genes found upon comparison of the 72h Ni^{2+}-exposed condition and the respective control treatment for the CD4$^+$ IL7R^{hi} clusters c0 (sheet 1) and cluster c2 (sheet 2) or the cytotoxic T cell cluster c3 (sheet3), respectively. Additionally, a summary list of uniquely or commonly regulated genes of the three individually compared T cell clusters is shown (sheet 4). Regulated genes signifying specific important memory T cell subsets as well as the commonly regulated genes KLF2 and its repressed target gene TOX [4] are highlighted by yellow shading (summary sheet 4) or white coloured font (individual sheets), respectively.

Supplementary References

1. Jin S, Guerrero-Juarez CF, Zhang L, et al. Inference and analysis of cell-cell communication using CellChat. *Nat Commun*. Feb 17 2021;12(1):1088. doi:10.1038/s41467-021-21246-9
2. Qiu X, Mao Q, Tang Y, et al. Reversed graph embedding resolves complex single-cell trajectories. *Nat Methods*. Oct 2017;14(10):979–982. doi:10.1038/nmeth.4402
3. Viemann D, Schmidt M, Tenbrock K, et al. The contact allergen nickel triggers a unique inflammatory and proangiogenic gene expression pattern via activation of NF-kappaB and hypoxia-inducible factor-1alpha. *J Immunol*. Mar 1 2007;178(5):3198–207. doi:10.4049/jimmunol.178.5.3198
4. Fagerberg E, Attanasio J, Dien C, et al. KLF2 maintains lineage fidelity and suppresses CD8 T cell exhaustion during acute LCMV infection. *Science*. Jan 2 2025;387(6735):eadn2337. doi:10.1126/science.adn2337

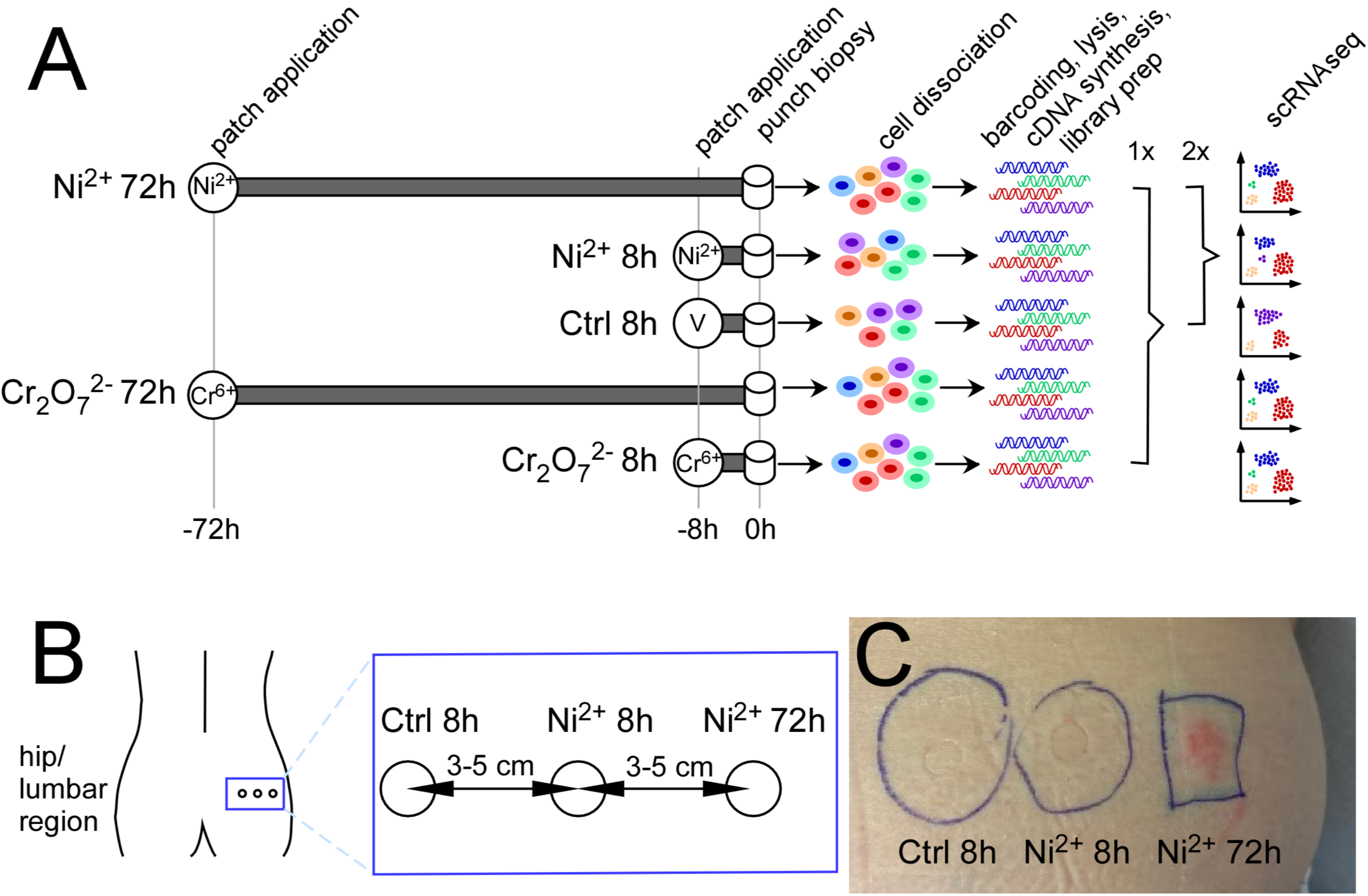

A
patch application
patch application
punch biopsy
cell dissociation
barcoding, lysis, cDNA synthesis, library prep
scRNAseq
1x
2x
Ni2+ 72h
Ni2+ 8h
Ctrl 8h
V
Cr2O72- 72h
Cr2O72- 8h
Cr6+
-72h
-8h
0h
B
hip/
lumbar
region
Ctrl 8h
Ni2+ 8h
Ni2+ 72h
3-5 cm
3-5 cm
C
Ctrl 8h
Ni2+ 8h
Ni2+ 72h

Figure S1

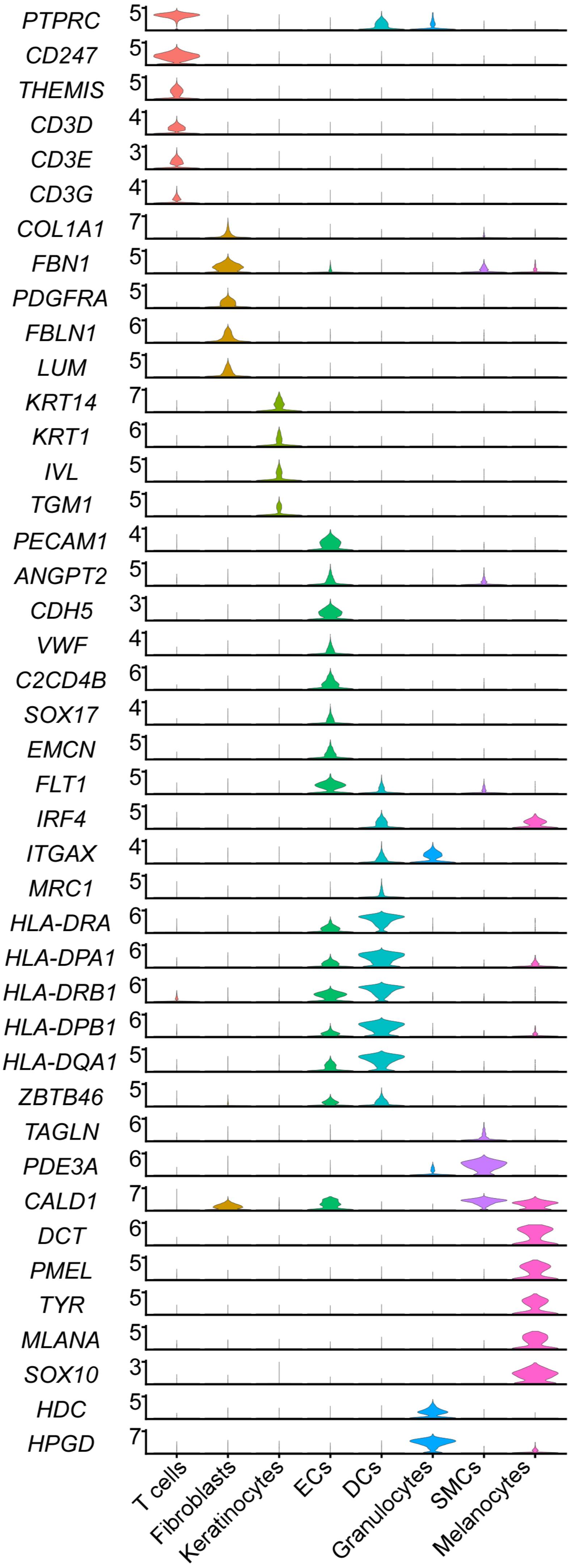

PTPRC
CD247
THEMIS
CD3D
CD3E
CD3G
COL1A1
FBN1
PDGFRA
FBLN1
LUM
KRT14
KRT1
IVL
TGM1
PECAM1
ANGPT2
CDH5
VWF
C2CD4B
SOX17
EMCN
FLT1
IRF4
ITGAX
MRC1
HLA-DRA
HLA-DPA1
HLA-DRB1
HLA-DPB1
HLA-DQA1
ZBTB46
TAGLN
PDE3A
CALD1
DCT
PMEL
TYR
MLANA
SOX10
HDC
HPGD
T cells
Fibroblasts
Keratinocytes
ECs
DCs
Granulocytes
SMCs
Melanocytes

Figure S2

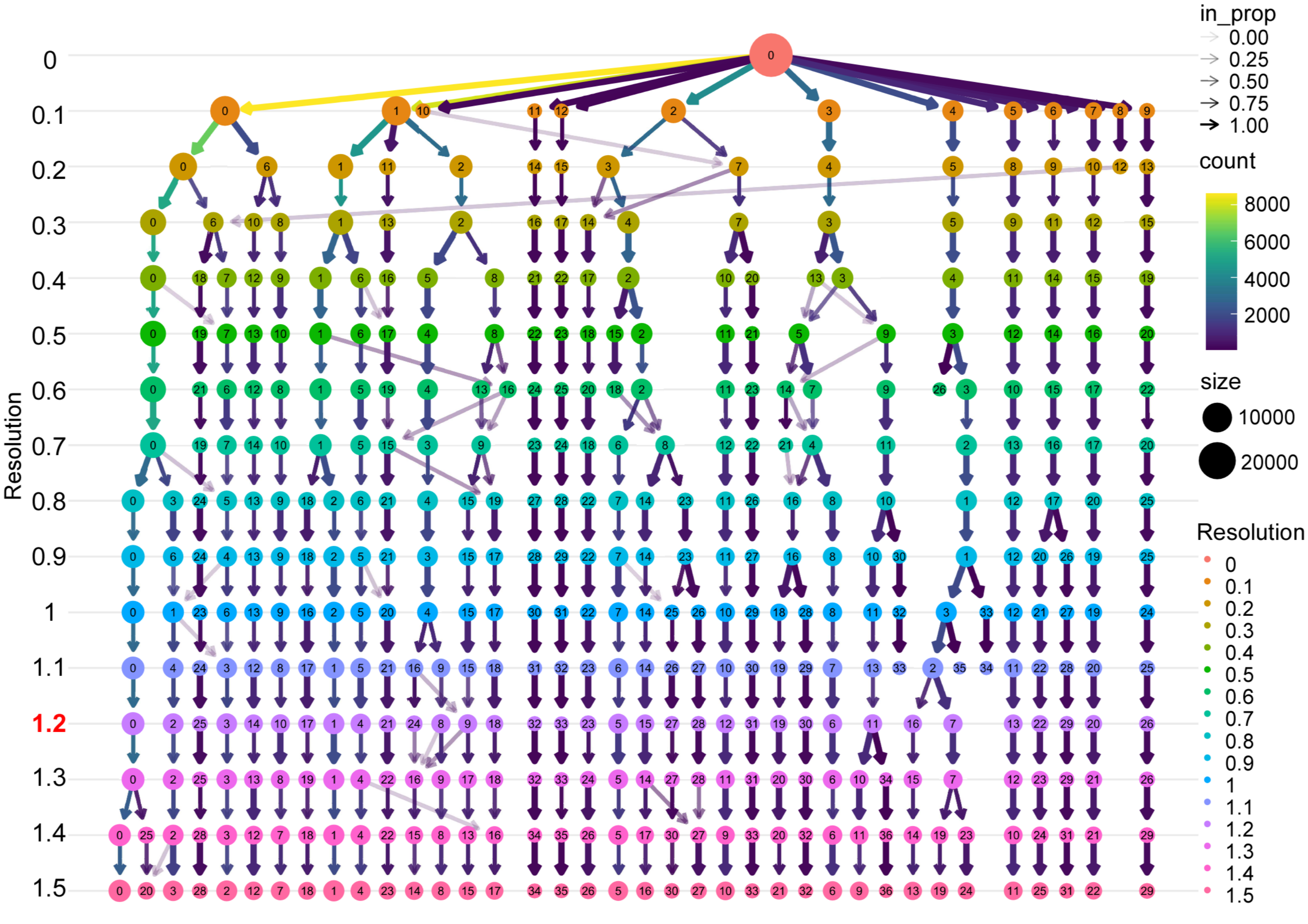
in_prop
0.00
0.25
0.50
0.75
1.00
count
8000
6000
4000
2000
size
10000
20000
Resolution
0
0.1
0.2
0.3
0.4
0.5
0.6
0.7
0.8
0.9
1
1.1
1.2
1.3
1.4
1.5
Resolution
0
0.1
0.2
0.3
0.4
0.5
0.6
0.7
0.8
0.9
1
1.1
1.2
1.3
1.4
1.5

Figure S3

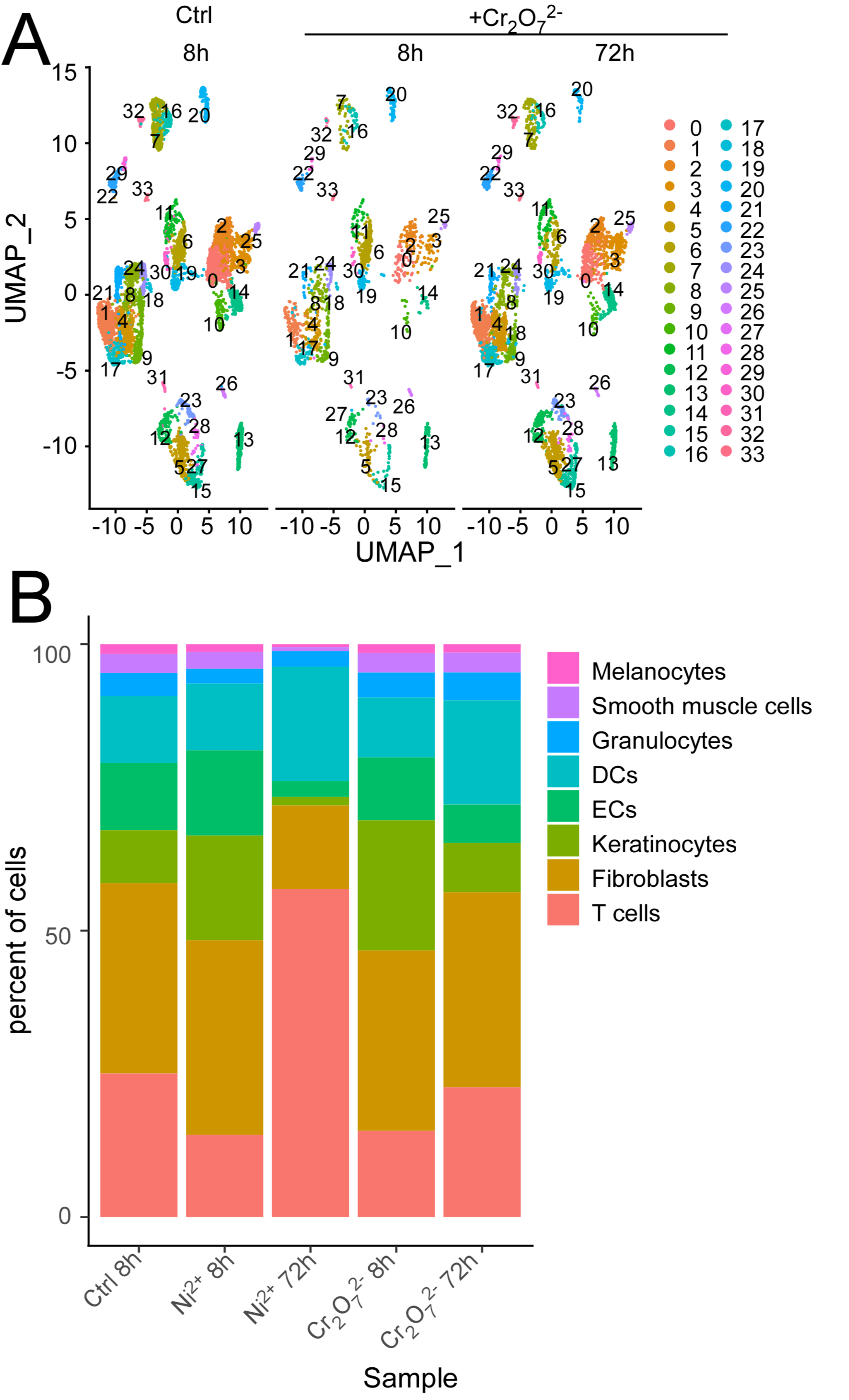
A
Ctrl
+$Cr_2O_7^{2-}$
8h
8h
72h
UMAP_2
UMAP_1
B
percent of cells
Melanocytes
Smooth muscle cells
Granulocytes
DCs
ECs
Keratinocytes
Fibroblasts
T cells
Ctrl 8h
Ni^{2+} 8h
Ni^{2+} 72h
$Cr_2O_7^{2-}$ 8h
$Cr_2O_7^{2-}$ 72h
Sample

Figure S4

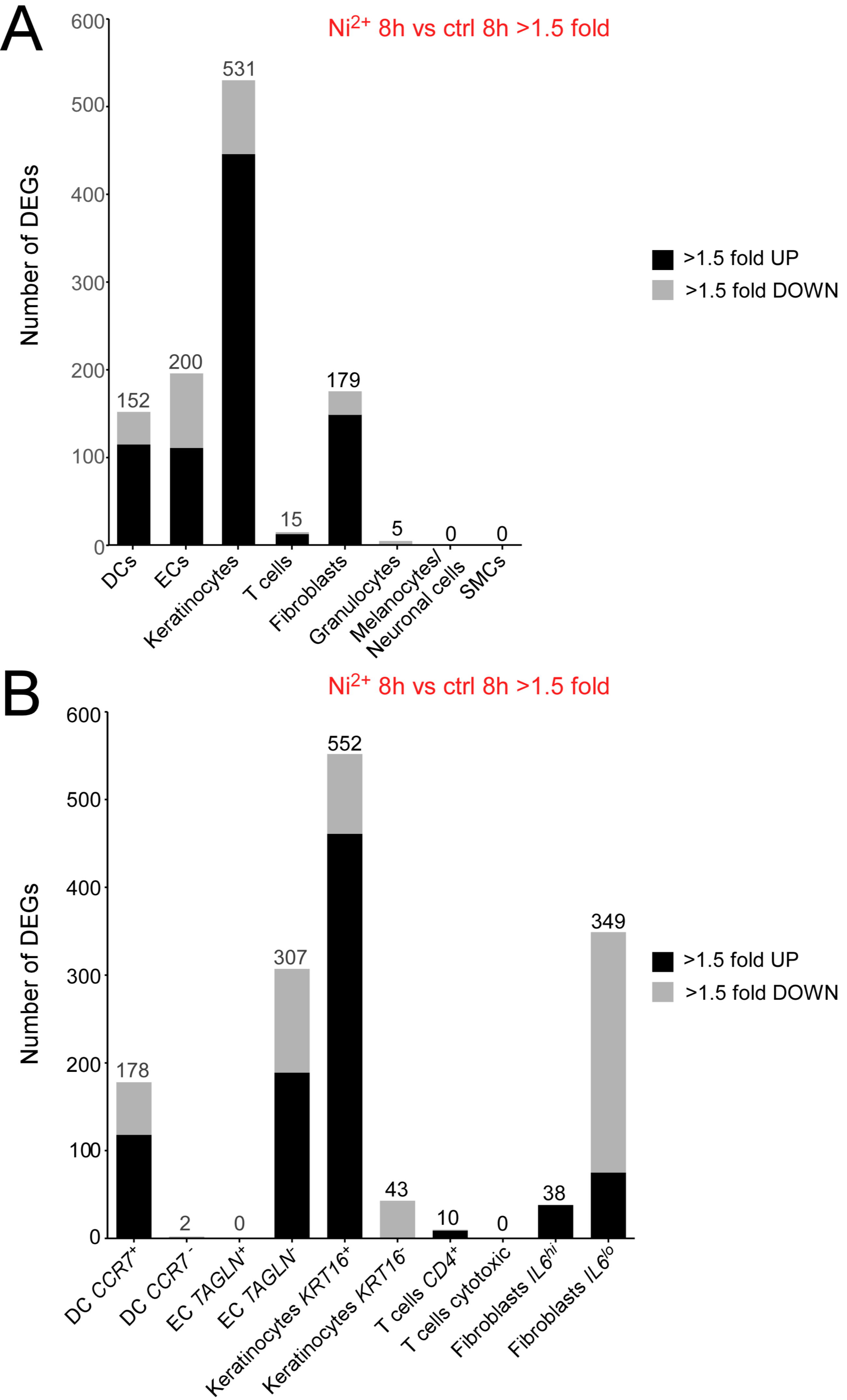
A
Ni2+ 8h vs ctrl 8h >1.5 fold
Number of DEGs
600
500
400
300
200
100
0
531
200
152
179
15
5
0
0
DCs
ECs
Keratinocytes
T cells
Fibroblasts
Granulocytes
Melanocytes/ Neuronal cells
SMCs
>1.5 fold UP
>1.5 fold DOWN
B
Ni2+ 8h vs ctrl 8h >1.5 fold
Number of DEGs
552
349
307
178
43
38
10
2
0
0
DC CCR7+
DC CCR7-
EC TAGLN+
EC TAGLN-
Keratinocytes KRT16+
Keratinocytes KRT16-
T cells CD4+
T cells cytotoxic
Fibroblasts IL6hi
Fibroblasts IL6lo
>1.5 fold UP
>1.5 fold DOWN

Figure S5

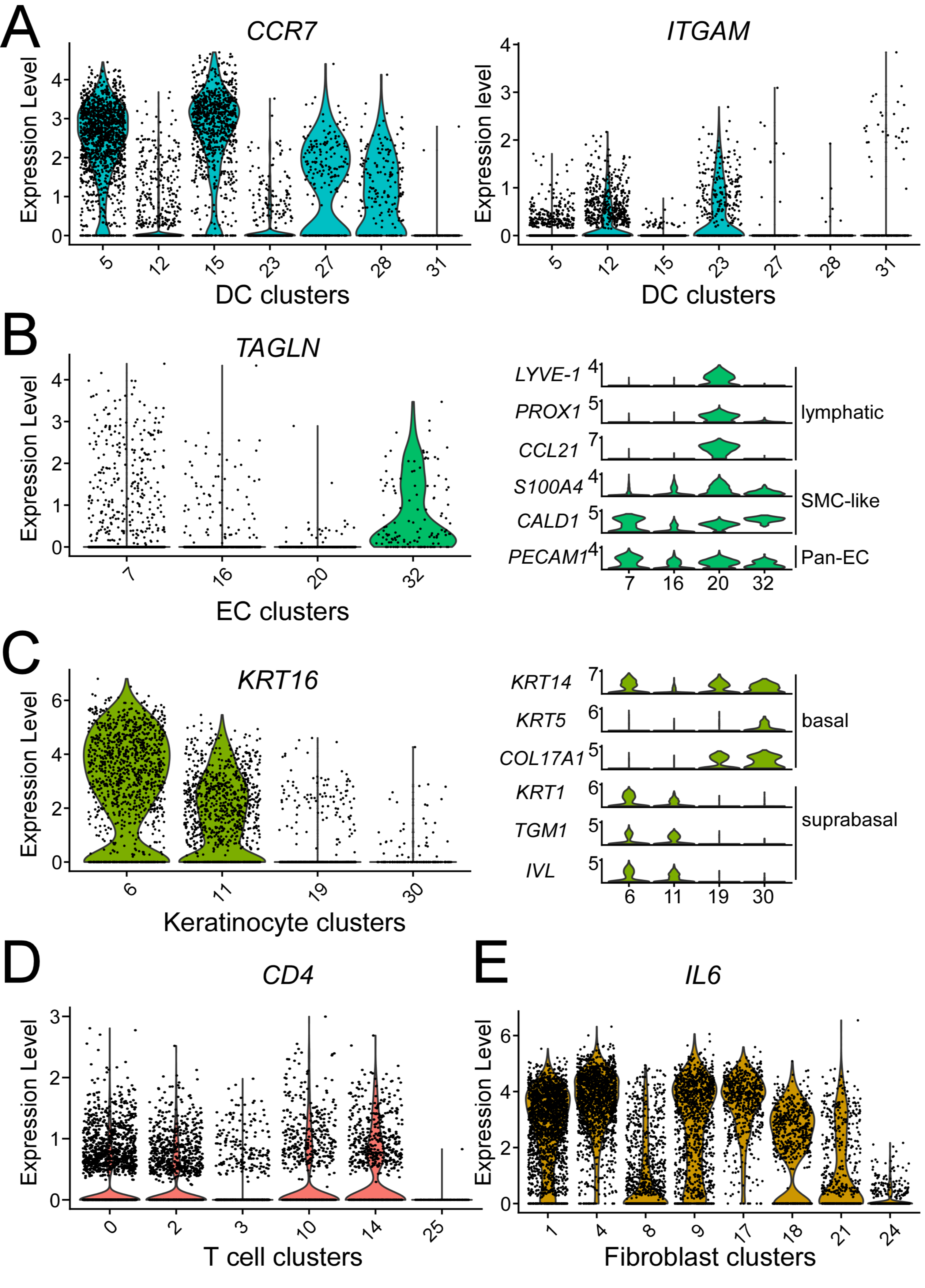
A
CCR7
Expression Level
DC clusters
5
12
15
23
27
28
31
ITGAM
Expression level
B
TAGLN
EC clusters
7
16
20
32
LYVE-1
PROX1
CCL21
S100A4
CALD1
PECAM1
lymphatic
SMC-like
Pan-EC
C
KRT16
Keratinocyte clusters
6
11
19
30
KRT14
KRT5
COL17A1
KRT1
TGM1
IVL
basal
suprabasal
D
CD4
T cell clusters
0
2
3
10
14
25
E
IL6
Fibroblast clusters
1
4
8
9
17
18
21
24

Figure S6

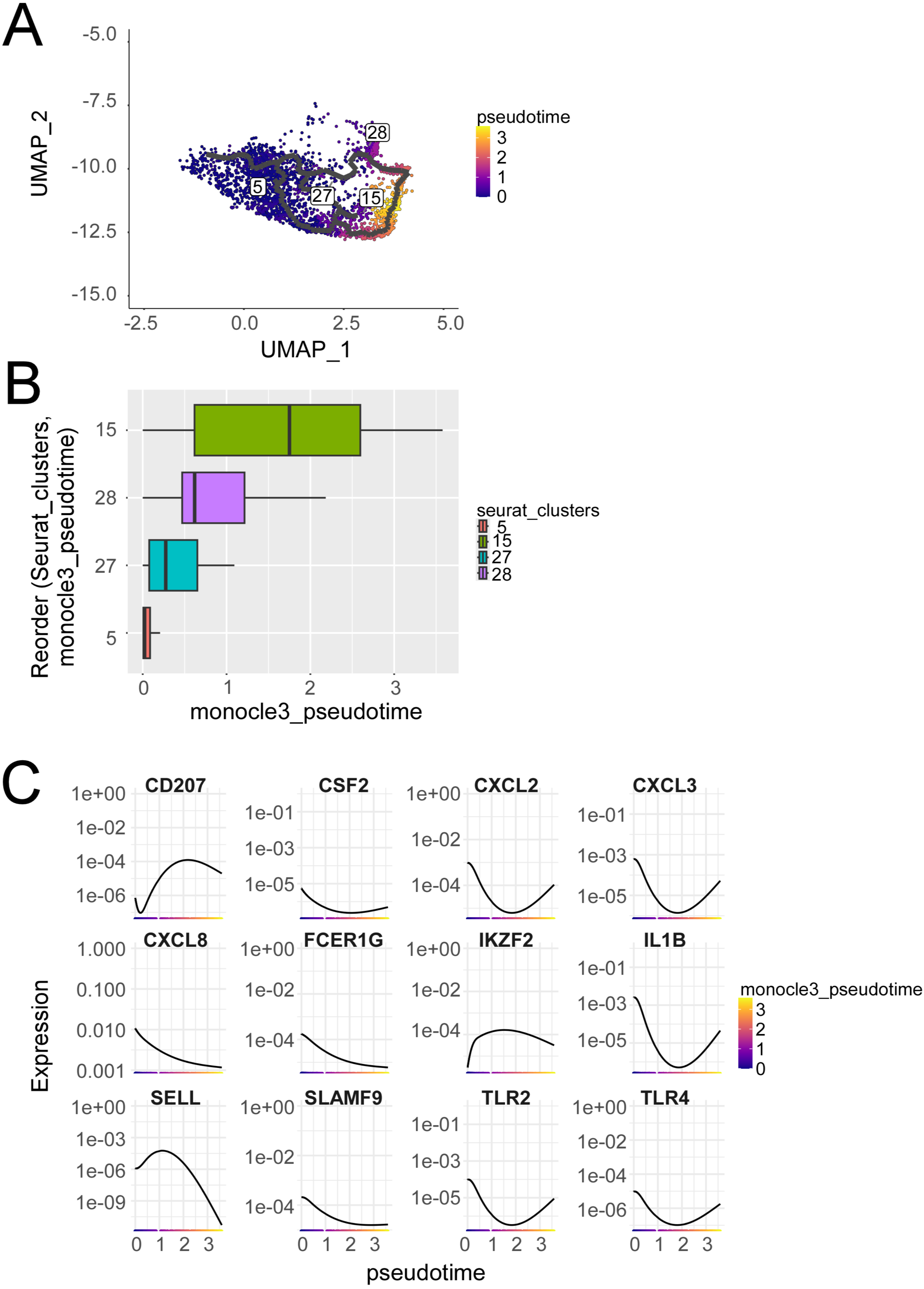
A
-5.0
-7.5
-10.0
-12.5
-15.0
UMAP_2
-2.5
0.0
2.5
5.0
UMAP_1
28
5
27
15
pseudotime
3
2
1
0
B
Reorder (Seurat_clusters, monocle3_pseudotime)
15
28
27
5
0
1
2
3
monocle3_pseudotime
seurat_clusters
5
15
27
28
C
CD207
CSF2
CXCL2
CXCL3
CXCL8
FCER1G
IKZF2
IL1B
SELL
SLAMF9
TLR2
TLR4
Expression
pseudotime
monocle3_pseudotime

Figure S7

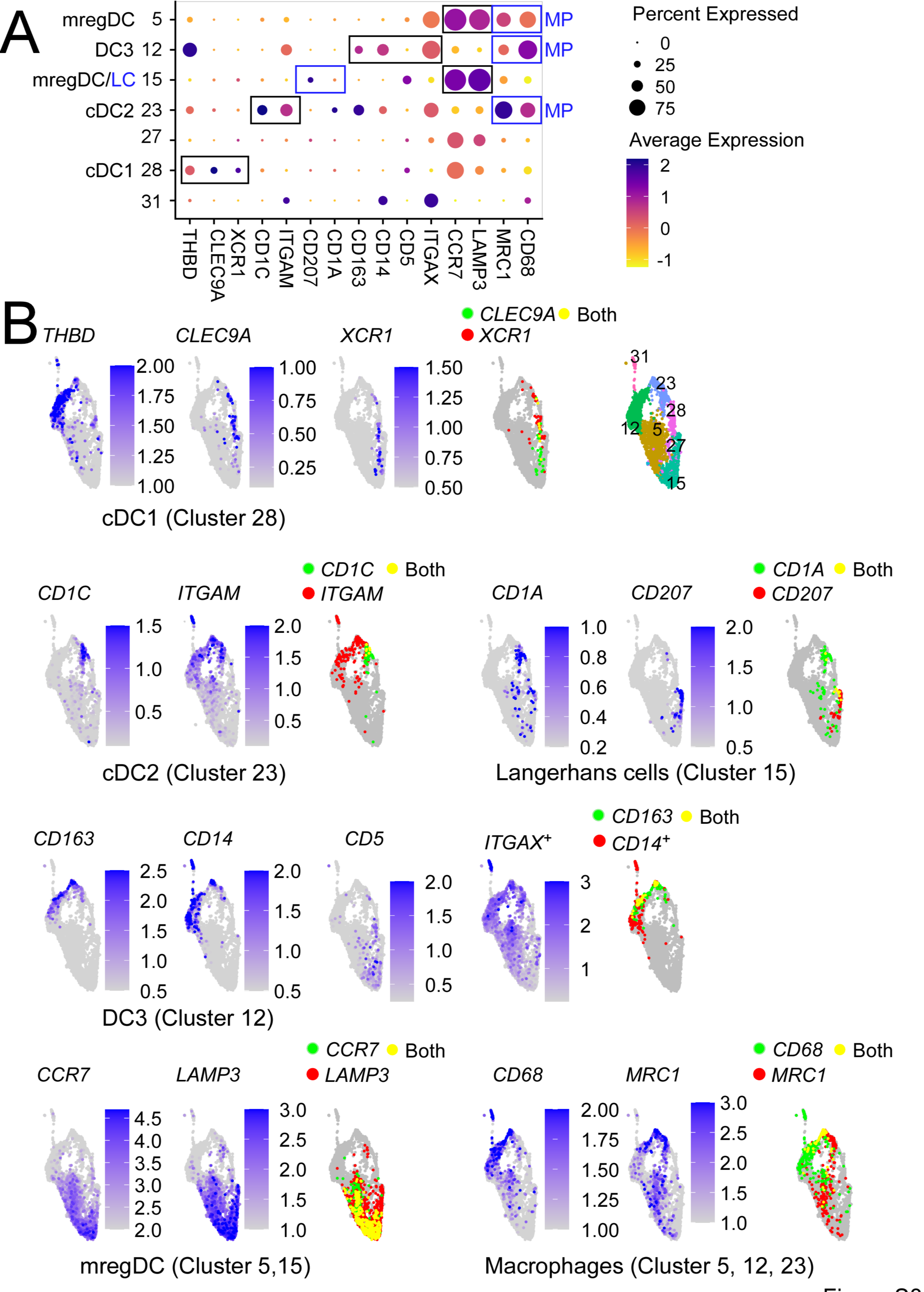
A
mregDC 5
DC3 12
mregDC/LC 15
cDC2 23
27
cDC1 28
31
MP
THBD
CLEC9A
XCR1
CD1C
ITGAM
CD207
CD1A
CD163
CD14
CD5
ITGAX
CCR7
LAMP3
MRC1
CD68
Percent Expressed
0
25
50
75
Average Expression
B
THBD
CLEC9A
XCR1
Both
cDC1 (Cluster 28)
CD1C
ITGAM
cDC2 (Cluster 23)
CD1A
CD207
Langerhans cells (Cluster 15)
CD163
CD14
CD5
ITGAX+
CD14+
DC3 (Cluster 12)
CCR7
LAMP3
mregDC (Cluster 5,15)
CD68
MRC1
Macrophages (Cluster 5, 12, 23)

Figure S8

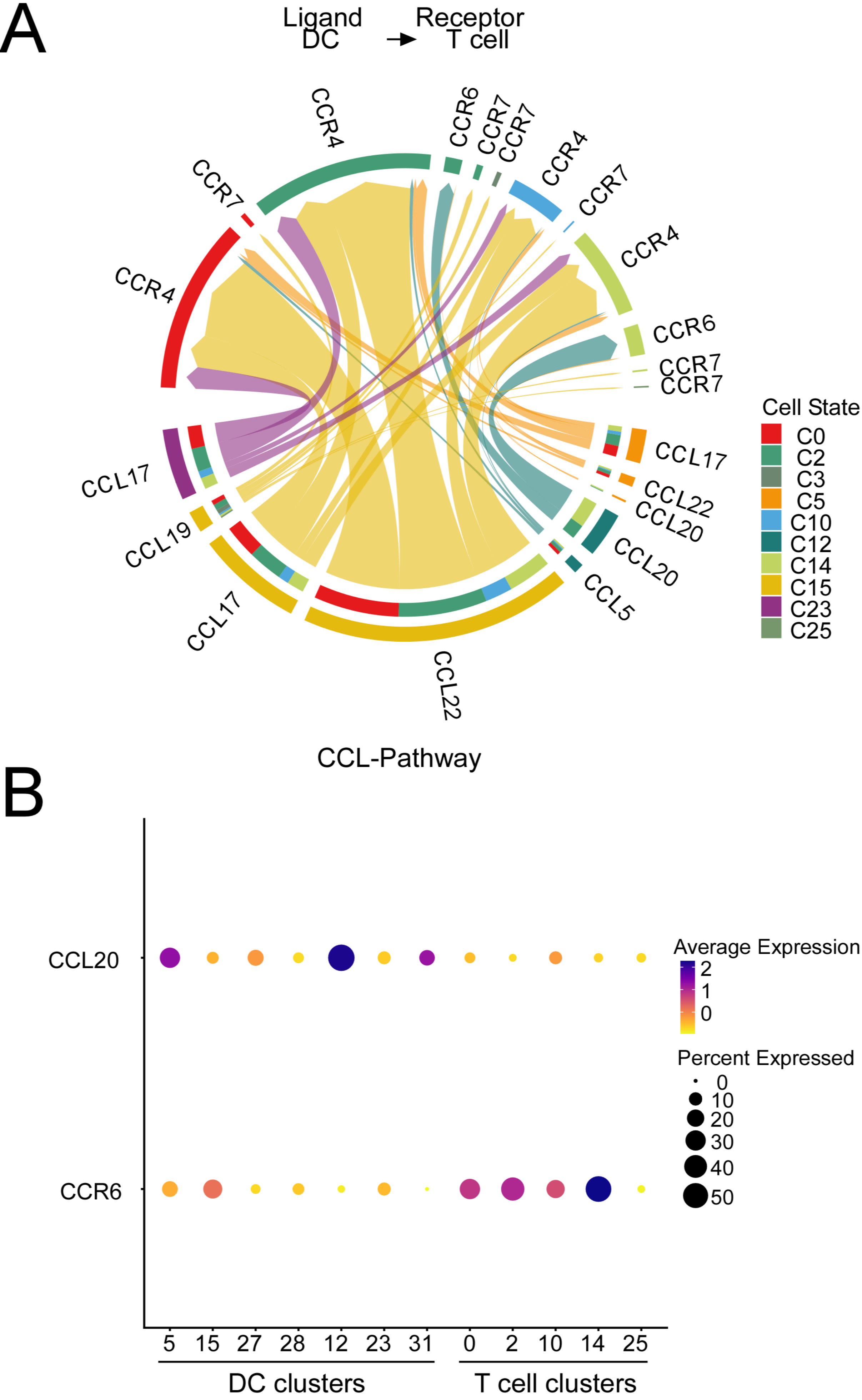
A
Ligand DC → Receptor T cell
CCR4
CCR6
CCR7
CCR7
CCR4
CCR7
CCR4
CCR6
CCR7
CCR7
CCL17
CCL22
CCL20
CCL20
CCL5
CCL22
CCL17
CCL19
CCL17
CCR4
CCR7
Cell State
C0
C2
C3
C5
C10
C12
C14
C15
C23
C25
CCL-Pathway
B
CCL20
CCR6
5 15 27 28 12 23 31 0 2 10 14 25
DC clusters
T cell clusters
Average Expression
2
1
0
Percent Expressed
0
10
20
30
40
50

Figure S9

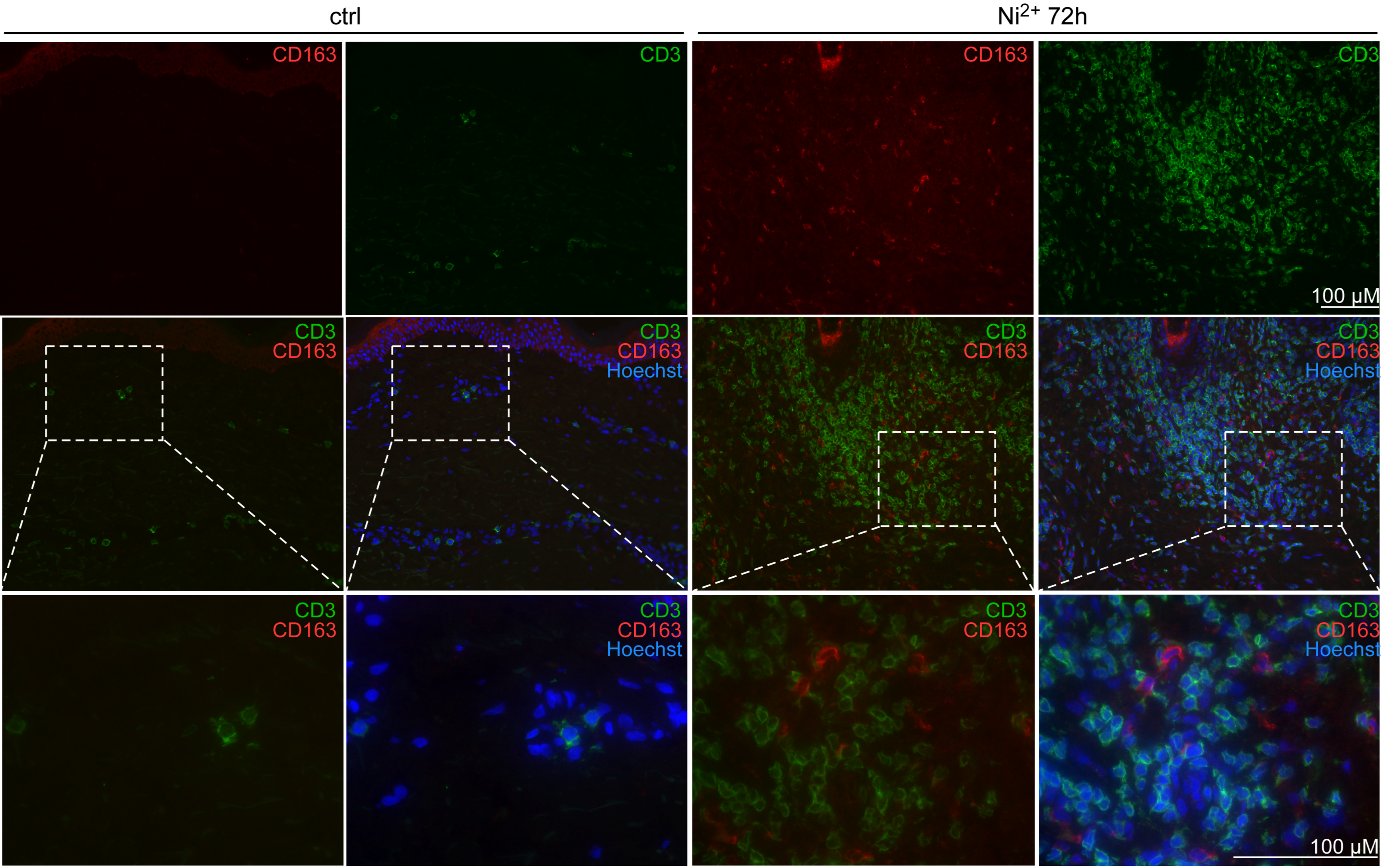
ctrl
Ni2+ 72h
CD163
CD3
CD163
CD3
100 μM
CD3
CD163
CD3
CD163
Hoechst
CD3
CD163
CD3
CD163
Hoechst
CD3
CD163
CD3
CD163
Hoechst
CD3
CD163
CD3
CD163
Hoechst
100 μM

Figure S10

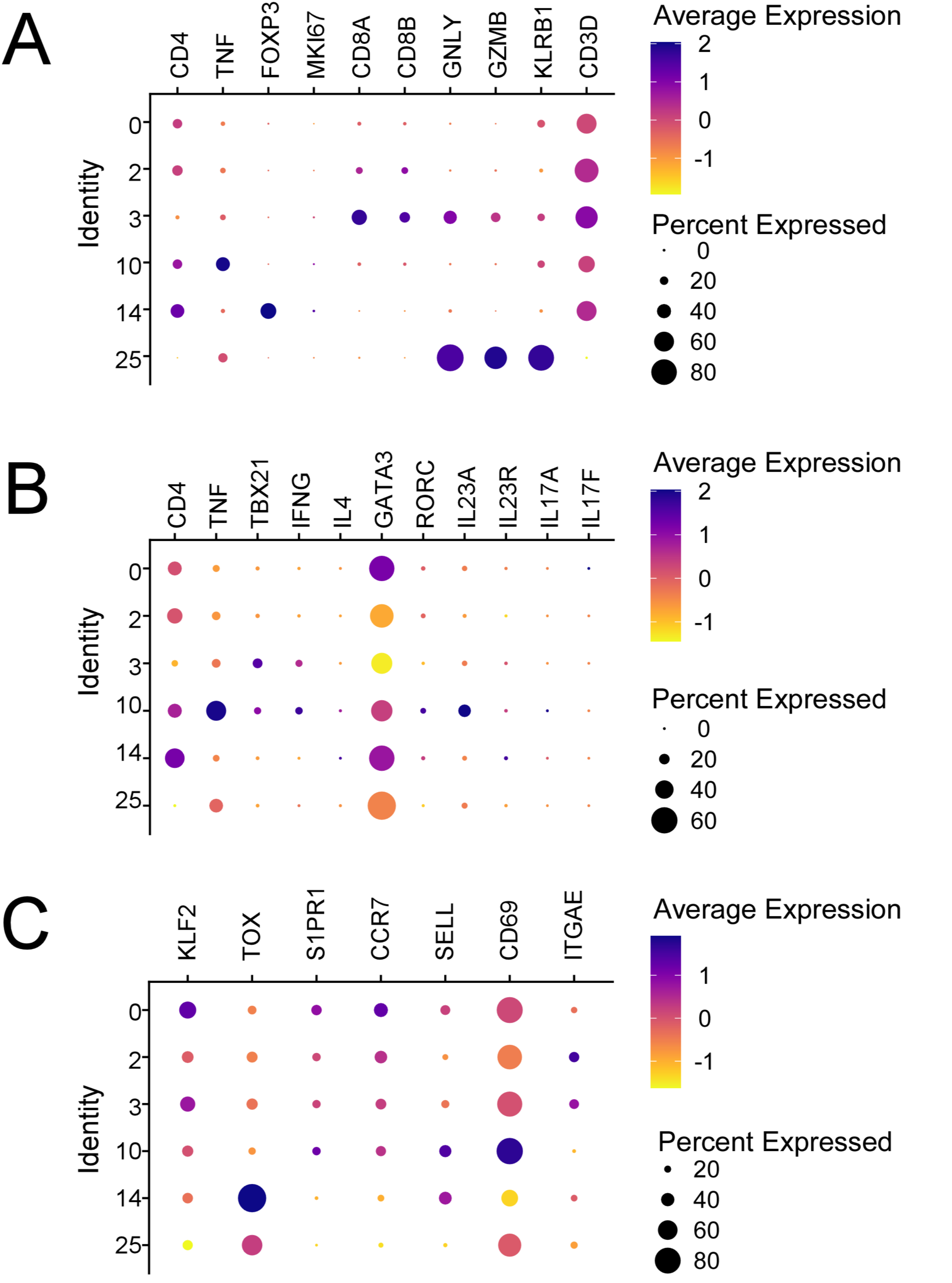
A
CD4
TNF
FOXP3
MKI67
CD8A
CD8B
GNLY
GZMB
KLRB1
CD3D
Identity
0
2
3
10
14
25
Average Expression
2
1
0
-1
Percent Expressed
0
20
40
60
80
B
CD4
TNF
TBX21
IFNG
IL4
GATA3
RORC
IL23A
IL23R
IL17A
IL17F
Identity
0
2
3
10
14
25
Average Expression
2
1
0
-1
Percent Expressed
0
20
40
60
C
KLF2
TOX
S1PR1
CCR7
SELL
CD69
ITGAE
Identity
0
2
3
10
14
25
Average Expression
1
0
-1
Percent Expressed
20
40
60
80

Figure S11

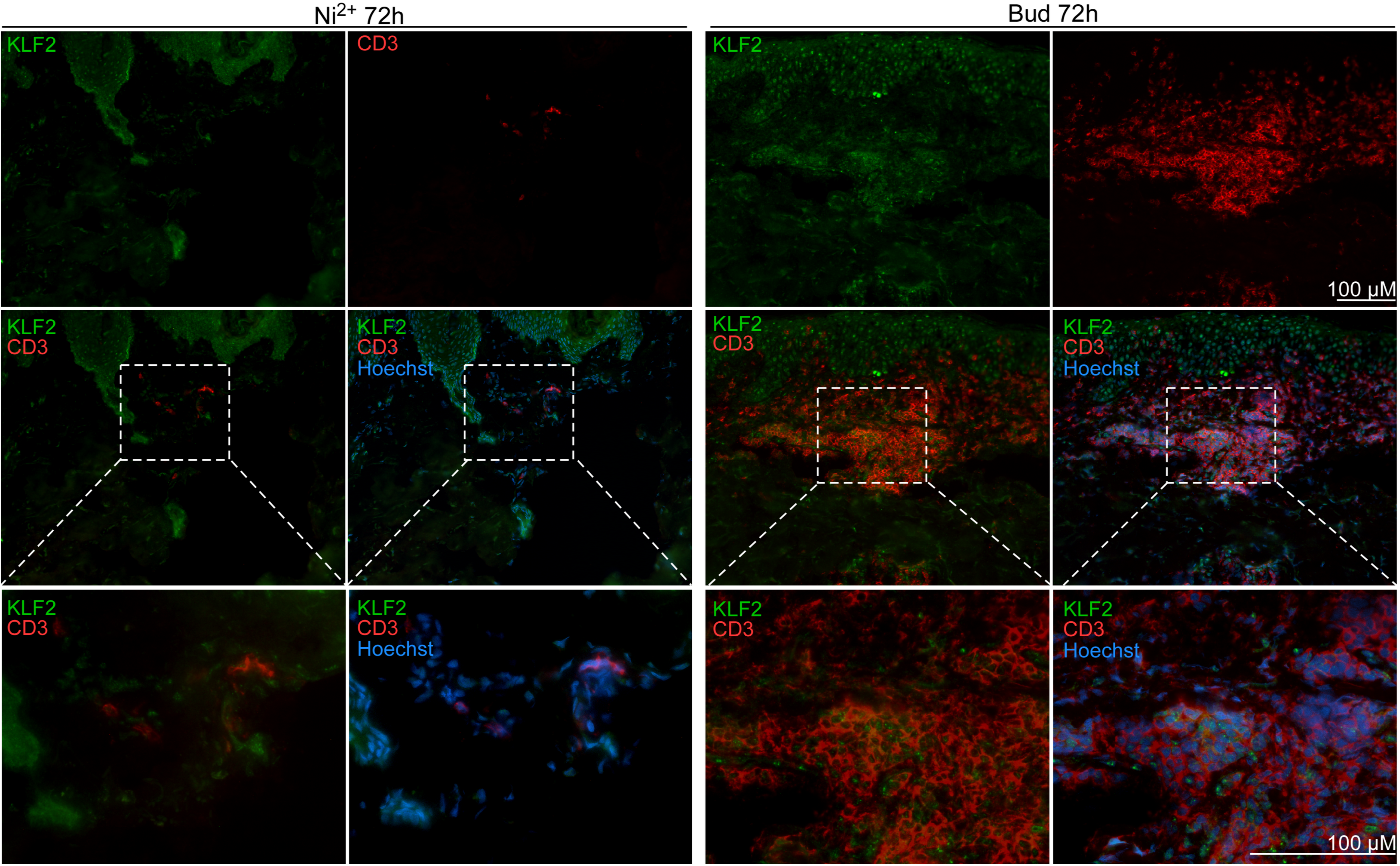

Ni2+ 72h
Bud 72h
KLF2
CD3
KLF2
100 μM
KLF2
CD3
KLF2
CD3
Hoechst
KLF2
CD3
KLF2
CD3
Hoechst
KLF2
CD3
KLF2
CD3
Hoechst
KLF2
CD3
KLF2
CD3
Hoechst
100 μM

Figure S12
